# Bio-Inspired PSO Optimization of Fuzzy Membership Boundaries and PI Gains for a 48 V Synchronous Boost Converter

**DOI:** 10.3390/biomimetics11090656

**Published:** 2026-09-12

**Authors:** Teoman Karadag, Emre Gozkaya, Arif Basgumus, Mustafa Namdar

**Affiliations:** 1 Department of Electrical and Electronics Engineering, Inonu University, 44280 Malatya, Türkiye; 2 Department of Electrical and Electronics Engineering, Istinye University, 34396 Istanbul, Türkiye; 3 Department of Electrical and Electronics Engineering, Bursa Uludag University, 16059 Bursa, Türkiye; 502405012@ogr.uludag.edu.tr or emre.gozkaya@ozdilek.com.tr (E.G.); basgumus@uludag.edu.tr (A.B.); 4 Ozveri R&D Center, Ozdilek Ev Tekstil Sanayi ve Ticaret A.S., 16150 Bursa, Türkiye; 5 Department of Electrical and Electronics Engineering, Kutahya Dumlupinar University, 43100 Kutahya, Türkiye; mustafa.namdar@dpu.edu.tr

**Keywords:** approximate reasoning, bio-inspired control, membership-boundary optimization, DC–DC converter, fuzzy logic control, synchronous boost converter, particle swarm optimization, swarm intelligence

## Abstract

48 V bus modules must stay regulated under wide input-voltage swings and fast load transients. A proportional-integral (PI) controller and a fuzzy logic controller (FLC) are tuned by the same Particle Swarm Optimization (PSO), a swarm metaheuristic modeled on bird-flock foraging, under an Integral of Time-weighted Absolute Error (ITAE) objective. The novelty is PSO-based membership-boundary optimization: the membership-function limits, analogues of biological decision thresholds, are shaped directly rather than only the scaling gains. A 153 W, 225 kHz synchronous boost converter is simulated with non-ideal parasitics. For input-voltage transitions the PSO-FLC shortens settling time by up to 70.5% (118 versus 400 ms), whereas the PSO-PI gives smaller peak deviation (26.2% versus 40.0%) and faster load-step recovery. Unconstrained, both controllers draw 58–62 A on the worst input-voltage step; a 10 A cycle-by-cycle limit removes these excursions. Under an identical budget and parameter count, boundary tuning attains 12.3% lower ITAE cost and roughly 40% shorter large-signal settling than scaling-gain tuning. Across ten runs the cost varies by under 1.5% of its mean, and the ranking holds at a realizable update rate (4.44 μs, zero-order hold) and under the current limit. The findings are simulation-only comparative design guidance.

## 1. Introduction

The increasing demand for power density and efficiency in transportation, industrial automation, and military electrical platforms has accelerated the adoption of 48 V bus architectures as an alternative to traditional 24 V/28 V systems. Lower distribution currents at 48 V reduce copper losses and ease thermal constraints, but they also necessitate high-performance direct-current-to-direct-current (DC–DC) converters that regulate the bus under wide input variations and fast load transients. Boost converters are a natural candidate when stepping up a lower input to a higher DC bus. However, they exhibit nonlinear dynamics and a right-half-plane zero (RHPZ) in continuous conduction mode (CCM), which complicates control design and limits achievable bandwidth. In addition, diode conduction loss becomes a major efficiency limitation in high-current applications. Synchronous rectification replaces the passive diode with a controlled metal-oxide-semiconductor field-effect transistor (MOSFET), significantly reducing conduction losses and improving thermal management.

Maintaining a stable output voltage in synchronous boost converters despite input voltage fluctuations, load variations, and parasitic component effects requires robust closed-loop control strategies. While traditional proportional-integral (PI) controllers are the industry standard due to their simplicity, their performance degrades when the converter’s operating point shifts. To overcome the limitations of linear controllers in nonlinear systems, fuzzy logic controllers (FLC) has gained prominence. FLC operates independently of exact mathematical models and offer robust, nonlinear control actions through heuristic rule bases, making them highly adaptable to dynamic variations.

Crucially, the fuzzy logic controller is itself a bio-inspired mechanism. Rather than manipulating precise numerical error signals in isolation, it emulates the way humans make decisions under uncertainty: perceptions are quantized into imprecise linguistic categories described by graded membership functions [1] (such as “negative,” “zero,” and “positive”), and actions are inferred through a base of if–then rules that mirror expert cognition [2]. In this study the FLC is therefore interpreted as a cognition-inspired approximate-reasoning layer that maps graded perceptions to graded control actions under uncertainty; it is not intended as a physiological model of neural information processing, and no claim of biological fidelity at the level of neurons or synapses is made. Framed this way, the behavior of the controller is governed by the geometry of its membership functions—the internal decision boundaries that translate perception into action.

However, determining the optimal PI gains ($K_{p}$, $K_{i}$) or fine-tuning FLC membership functions via trial-and-error often prevents the system from reaching its maximum dynamic potential. Consequently, metaheuristic optimization algorithms have been widely adopted in power electronics. Particle Swarm Optimization (PSO) is particularly appealing due to its structural simplicity, derivative-free nature, and rapid convergence on the class of low-dimensional, non-convex tuning problems considered here [3,4,5,6].

PSO is, at its origin, a direct transfer of biological collective behavior into a computational optimizer [4]. It abstracts the social foraging of biological swarms—flocking birds and schooling fish—in which no single individual possesses a global map, yet the group converges efficiently on the richest region by combining personal memory with information shared across the collective. Each artificial “particle” plays the role of a foraging animal, and the swarm’s cooperative search is what steers the population toward the optimum. Employing PSO to shape a fuzzy controller therefore couples two complementary layers of biomimicry: a population-level swarm search that adapts a cognition-level fuzzy reasoner. Early applications of PSO in DC-DC converters primarily focused on Maximum Power Point Tracking (MPPT) in photovoltaic systems, where PSO-tuned proportional-integral-derivative (PID) controllers demonstrated superior transient responses to conventionally tuned designs [7,8], and where swarm methods were applied to global maximum-power-point tracking and to comparisons against fuzzy and perturb-and-observe schemes [9,10,11]. Subsequent research expanded to complex topologies, such as single-ended primary-inductor converters (SEPIC) for power factor correction [12], Cuk converters [11], and robust PID designs for general power-converter stages [13], indicating the adaptability of swarm intelligence across topologies; fuzzy-controlled three-level boost stages have been explored in the same application area without a metaheuristic layer [14]. A parallel line of work combined metaheuristics with fractional-order and fuzzy structures, including PSO- and moth-flame-optimized fractional-order fuzzy PID controllers for boost converters [15], antlion-optimized fractional-order fuzzy PID design for buck converters [16], and PSO-tuned fractional fuzzy PI control for power factor improvement [17]. Swarm-based tuning has likewise been applied to fuel-cell and photovoltaic power stages, covering interleaved boost converters for fuel-cell voltage regulation [18], proton-exchange membrane (PEM) fuel-cell emulators and boost converters with PSO-tuned PI control [19,20], comparative grey wolf optimizer (GWO) and PSO-MPPT strategies [21], and parallel PSO implementations for synchronous constant-current converters [22].

In recent years, hybrid and robust control strategies have continued to expand the application of PSO in converter control. PSO has been used not only for high-accuracy PI tuning in DC-DC converters [23,24], but also for comparative evaluations between PI- and fuzzy-based control structures in buck–boost and related converter topologies [25,26]. In parallel, fuzzy and hybrid fuzzy control strategies have been explored in high step-up and fuel-cell-related converter applications, demonstrating improved adaptability under nonlinear operating conditions [27,28]. More recent studies have further extended this direction by comparing PI-PSO, fuzzy-PID, and adaptive fuzzy approaches [29], as well as by examining PSO-based stability behavior in buck–boost converters through simulation-oriented analysis [30].

Despite these extensive advancements, a critical limitation persists in the current state-of-the-art. Many recent metaheuristic FLC studies primarily tune controller gains or external scaling factors while retaining fixed membership geometries [31,32]. In such approaches, the internal structure of the fuzzy controller remains static. Although recent work has begun exploring membership parameterization in DC–DC stages [33], and related studies have investigated the parameterization of fuzzy controllers more broadly [34], direct structural optimization of internal decision boundaries alongside comparative linear baselines under identical swarm budgets remains an open area of investigation. Swarm and swarm-hybrid metaheuristics continue to be applied to boost-converter controller design [35] and to constrained engineering optimization problems in general [36], but without such a controlled internal comparison. Therefore, this paper focuses on a comprehensive, fair comparative study of two PSO-optimized control strategies for a 153 W synchronous boost converter operating under a wide input range (22–30 V) to a 48 V bus. Both a PSO-tuned PI controller and a PSO-optimized FLC are tuned using the exact same Integral of Time-weighted Absolute Error (ITAE) objective and evaluated under identical, simulation-based test scenarios.

The main contributions of this paper are as follows:

- The formulation of a bio-inspired, PSO-based membership-boundary optimization framework for FLC, in which the swarm-intelligence metaheuristic tunes the key membership-function limits (zero-region width and saturation threshold) that constitute the controller’s decision boundaries, rather than only the external scaling gains;
- A detailed model was developed in MATLAB Simulink R2024b, incorporating parasitic components (equivalent series resistance, ESR; direct-current resistance, DCR) and MOSFET switching losses for a realistic representation of the converter.;
- A rigorous comparative evaluation under large-signal input voltage steps, load transitions, and reference tracking scenarios;
- An ablation under an identical optimization budget that separates the contribution of membership-boundary tuning from that of conventional scaling-gain tuning, and from a three-parameter partial joint search that also releases the output scaling gain;
- A quantitative analysis exposing the key trade-off: faster large-signal adaptation with PSO-FLC versus tighter transient voltage containment with PSO-PI.

The remainder of this paper is organized as follows: Section 2 details the materials and methods, including the synchronous converter design, mathematical modeling, and the proposed bio-inspired PSO optimization framework. Section 3 presents the comparative simulation results. Section 4 discusses the dynamic performance trade-offs and states the limitations of the study, and Section 5 concludes the paper.

## 2. Materials and Methods

This section presents the synchronous boost converter model, the bio-inspired PSO-based controller design procedure, and the detailed non-ideal switching model adopted for the comparative analysis.

### 2.1. Converter Design and Modeling

#### 2.1.1. Topology and Key Specifications

**Figure 1:**
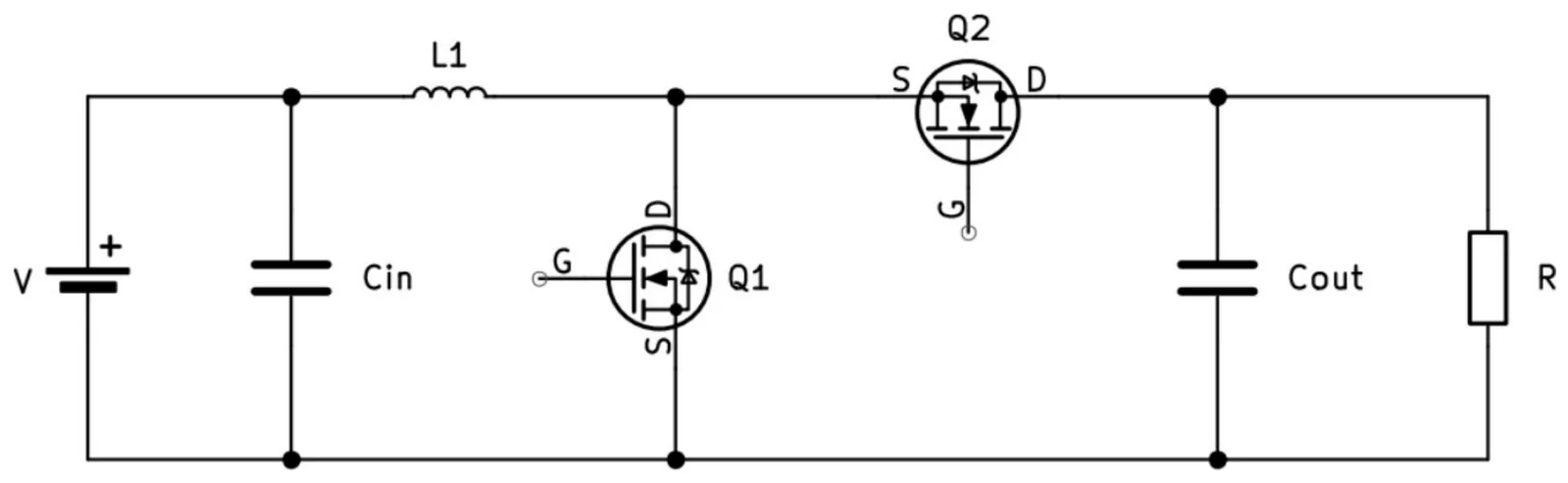
Synchronous boost converter topology (main switch Q1 and synchronous rectifier Q2).

Figure 1 shows the synchronous boost converter topology used in this study. The investigated converter is designed for $P_{out}\approx 153$ W with $f_{sw}=225$ kHz and the ranges $V_{in}\in \left[22,30\right]$ V and $V_{out}\in \left[45,55\right]$ V. Under the ideal CCM boost relationship [37],

(1) $$V_{out}=\frac{V_{in}}{1-D},\;D=1-\frac{V_{in}}{V_{out}},$$

which yields $D_{min}\approx 0.333$ (30 V → 45 V) and $D_{max}\approx 0.60$ (22 V → 55 V). Table 1 summarizes the key parameters used in simulation.

#### 2.1.2. Averaged CCM Model

Let the state variables be the inductor current $i_{L}$ and the output capacitor voltage $V_{out}$. Neglecting switching dynamics, the converter has two switching sub-intervals.

ON interval (main switch ON, synchronous MOSFET OFF):

(2) $$\frac{di_{L}}{dt}=\frac{V_{in}}{L},\;\frac{dV_{out}}{dt}=-\frac{i_{o}}{C_{out}},$$

OFF interval (main switch OFF, synchronous MOSFET ON):

(3) $$\frac{di_{L}}{dt}=\frac{V_{in}-V_{out}}{L},\;\frac{dV_{out}}{dt}=\frac{i_{L}-i_{o}}{C_{out}},$$

Using duty ratio *D* and averaging over one switching period $T_{sw}$ gives the CCM averaged model:

(4) $$\frac{di_{L}}{dt}=\frac{V_{in}-\left(1-D\right)V_{out}}{L},$$

(5) $$\frac{dV_{out}}{dt}=\frac{\left(1-D\right)i_{L}-i_{o}}{C_{out}},$$

These equations highlight the nonlinearity through $\left(1-D\right)V_{out}$ and $\left(1-D\right)i_{L}$ terms.

### 2.2. Bio-Inspired PSO-Based Controller Optimization

#### 2.2.1. PSO Algorithm and Objective

PSO is a population-based, bio-inspired stochastic optimization method. It abstracts the collective intelligence that emerges when biological swarms—such as bird flocks and fish schools—forage cooperatively: no individual holds a global map, yet the group converges efficiently on the richest region by sharing information. Each particle represents a candidate solution vector $x_{i}$ and has a velocity $v_{i}$. At iteration *k*, the update equations are

(6) $$v_{i}^{k+1}=wv_{i}^{k}+c_{1}r_{1}\left(pbest_{i}-x_{i}^{k}\right)+c_{2}r_{2}\left(gbest-x_{i}^{k}\right),$$

(7) $$x_{i}^{k+1}=x_{i}^{k}+v_{i}^{k+1},$$

where *w* is inertia, $c_{1}$ and $c_{2}$ are cognitive and social coefficients, and $r_{1},r_{2}\in \left[0,1\right]$. The biomimetic core of the algorithm lies in these two coefficients: the cognitive term (pbest) rewards each particle’s own best remembered experience, while the social term (gbest) draws it toward the best experience shared across the swarm—directly mirroring how individual memory and social communication jointly steer a foraging collective. The same ITAE cost function is used to tune both controller types:

(8) $$J_{ITAE}=\int_{0}^{T}t\;\left|e\left(t\right)\right|\;dt,$$

where $J_{ITAE}$ is the objective value, $e\left(t\right)=V_{ref}\left(t\right)-V_{out}\left(t\right)$ is the output-voltage tracking error, *t* is time, and *T* is the total evaluation horizon. This objective penalizes persistent errors and encourages fast settling with small steady-state error, but does not directly constrain peak transient overshoot.

To ensure robust performance across varying operating conditions and to prevent the controllers from overfitting to a single steady-state point, a comprehensive dynamic test scenario was simulated during each PSO evaluation phase. Specifically, the ITAE cost function in Equation (8) was evaluated over a total simulation time of T=2.5 s. Rather than relying solely on a simple start-up response, the evaluation profile subjected the synchronous boost converter to a sequence of large-signal transient events. The scenario initiated with a start-up phase aiming for the nominal 48 V reference under a constant 24 V input. Subsequently, programmed disturbances were introduced into the simulation loop: a load step from 1.6 A to 3.19 A at t=1.0 s, followed by an input-voltage transition within the 22–30 V operating range. The reference was held at 48 V throughout the tuning run, so the reference-tracking scenario of Section 2.3.3 was not part of the optimization profile. By continuously accumulating the time-weighted absolute error across these sequential start-up and disturbance phases, the PSO algorithm was driven to identify parameter sets that favor rapid tracking and strong disturbance rejection under a common comparison criterion.

To account for the stochastic nature of the PSO algorithm, ten independent optimization runs were executed for each controller. A fixed swarm size of 20 particles and a maximum of 100 iterations were used for every run. The PSO parameters adopted in this study are summarized in Table 2.

These runs used the same swarm size, iteration limit, objective function and search bounds, with the seed sequence fixed at 101–110 for reproducibility. The per-run parameter sets, the attained ITAE values and their mean, median and sample standard deviation are reported in Table 3. Both searches exhibit a low dispersion of the attained cost, with sample standard deviations of σ=0.0446 for PSO-PI and σ=0.0425 for PSO-FLC, corresponding to approximately 1.1% and 1.4% of the respective means.

A Mann–Whitney rank-sum test on the two sets of ten attained costs rejects equality of the searches (U=100, p=0.0002, two-sided); the samples do not overlap, the highest PSO-FLC cost lying below the lowest PSO-PI cost. The parameter sets attaining the minimum final ITAE cost among the ten runs ($K_{p}=0.7778,K_{i}=19.7972$ for PI in Run 3; $ZE_{width}=7.514\;V$, $PB_{sat}=20.756\;V$ for FLC in Run 6) were selected for the detailed time-domain dynamic comparisons presented in Section 3.

These specific PSO parameters ($c_{1}=1.5$, $c_{2}=1.5$, and w=0.7) lie in the range commonly adopted in the converter-tuning literature cited in Section 1 [3,17,23]. This configuration was selected to provide a practical balance between global exploration and local exploitation while maintaining a reasonable computational burden.

**Figure 2:**
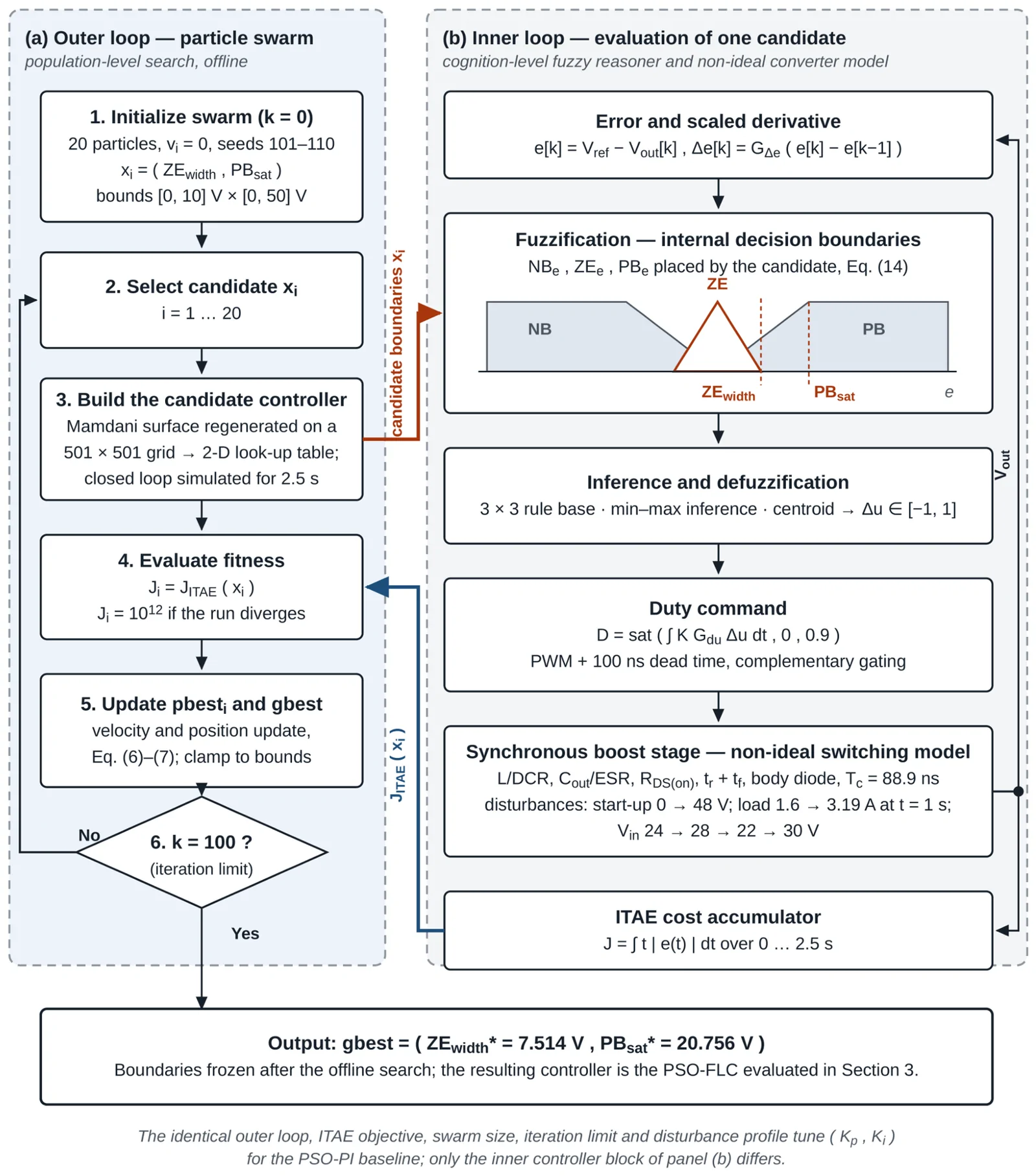
Proposed bio-inspired optimization framework combining the outer PSO search with inner FLC evaluation. (**a**) Offline PSO tuning of the voltage-error membership boundaries ($ZE_{width},PB_{sat}$); (**b**) closed-loop evaluation of each candidate using the non-ideal synchronous boost converter model. The same PSO loop, objective, and budget are used for the PSO-PI baseline and partially joint-optimized FLC variants in Section 3.6, with only the parameter vector $x_{i}$ and controller block differing. The ellipsis (…) denotes the index range, and the superscript * indicates the PSO-optimal parameter values.

The interaction between the population-level swarm search and the individual cognition-level fuzzy reasoning evaluation is detailed in the optimization framework presented in Figure 2.

As depicted, the outer loop in panel (a) manages particle velocity and position updates according to Equations (6) and (7), searching the two-dimensional boundary space ($ZE_{width},PB_{sat}$). For each candidate vector $x_{i}$, the inner loop in panel (b) regenerates the Mamdani control surface, executes the 2.5 s dynamic closed-loop simulation on the switch-level converter model incorporating non-ideal parasitics, and computes the accumulated ITAE cost $J_{i}$.

**Figure 3:**
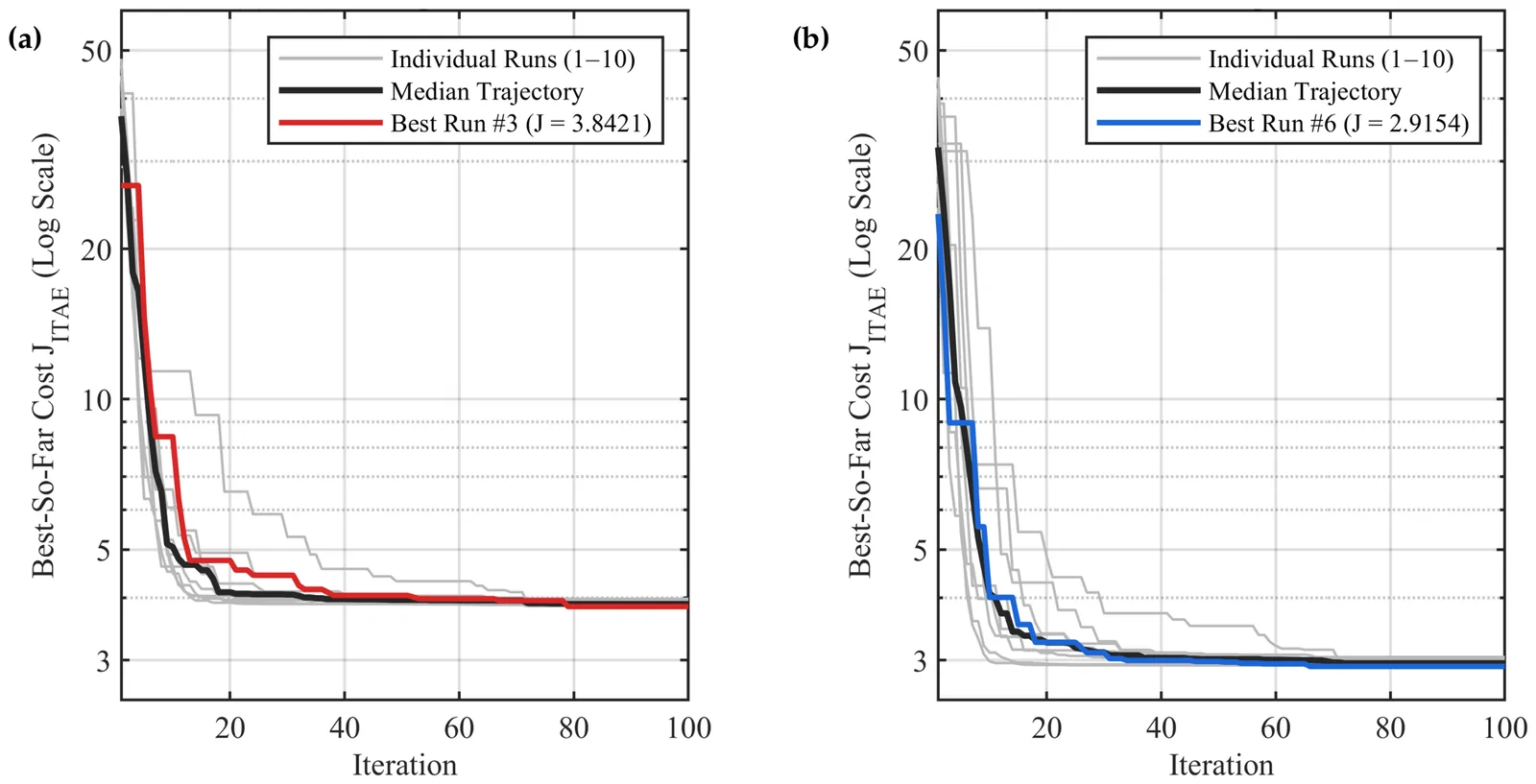
Convergence of the best-so-far ITAE cost over the ten independent PSO runs of the fixed seed sequence 101–110, on a logarithmic vertical scale. (**a**) PSO-PI search over $\left(K_{p},K_{i}\right)$; (**b**) PSO-FLC search over $\left(ZE_{width},PB_{sat}\right)$. Grey traces are the individual runs, the black trace is the per-iteration median across the ten runs, and the coloured trace is the run attaining the lowest final cost, whose parameters are used throughout Section 3.

The corresponding best-so-far cost trajectories are shown in Figure 3. Both searches complete the greater part of their descent within the first fifteen iterations, reducing the cost by roughly an order of magnitude relative to the initial swarm, and reach the immediate neighbourhood of their final value by about the thirtieth iteration; the remaining iterations produce only small refinements and every trajectory is flat well before the 100-iteration limit of Table 2, so that limit is not the binding constraint on the reported solutions. The ten trajectories of each controller collapse into a narrow band, which is the trajectory-level counterpart of the small final-cost dispersion in Table 3. The two final-cost distributions are moreover disjoint—the lowest PSO-PI cost, 3.8421, exceeds the highest PSO-FLC cost, 3.0447—so the ranking of the two parameterizations does not depend on which run is selected for the time-domain comparison.

#### 2.2.2. PSO-Tuned PI Controller

The continuous-time PI controller is

(9) $$G_{PI}\left(s\right)=K_{p}+\frac{K_{i}}{s},$$

where $G_{PI}\left(s\right)$ denotes the continuous-time transfer function of the PI controller, $K_{p}$ is the proportional gain, $K_{i}$ is the integral gain, and *s* is the Laplace variable.

For discrete implementation, the incremental form can be expressed as

(10) $$u\left[k\right]=u\left[k-1\right]+K_{p}\left(e[k]-e[k-1]\right)+K_{i}T_{c}\;e\left[k\right],$$

where u[k] is the discrete control signal (duty-cycle command), $e\left[k\right]=V_{ref}\left[k\right]-V_{out}\left[k\right]$ is the sampled voltage error, and $T_{c}$ is the control-update interval used in the simulation. In the present model the discrete controller blocks inherit the solver step, so that $T_{c}=88.9$ ns, i.e., the control law is re-evaluated at every integration step and fifty times per switching period. This is a property of the numerical model and not a hardware execution period: the simulated controller is effectively continuous-time rather than a rate-limited digital implementation, and the consequences of this choice are discussed in Section 4.6.

To ensure a fair and stable optimization, a bounded search space is enforced. The search boundaries for the PI gains were constrained to $K_{p}\in \left[0,20\right]$ and $K_{i}\in \left[0,100\right]$. After completing the multiple optimization runs, the best-performing gains obtained in this study are $K_{p}=0.7778$ and $K_{i}=19.7972$.

#### 2.2.3. Bio-Inspired PSO-Tuned Fuzzy Logic Controller

Structure: The FLC is the bio-inspired core of the proposed control scheme, emulating human approximate reasoning. It utilizes two inputs: the error (*e*) and its change (Δe). In this study, a compact 3×3 rule base with three linguistic levels—negative big (NB), zero (ZE), and positive big (PB)—is intentionally selected for each input. These linguistic labels play the role of the perceptual categories a human operator would use, and keeping their number small mirrors the cognitive economy of expert decision-making. By maintaining a lean 3×3 architecture, the swarm-intelligence framework can more effectively focus on optimizing the key membership-function boundaries without unnecessarily expanding the search space. Mamdani min–max inference [2] and centroid defuzzification are applied, and the output scaling gain ($G_{du}$) is fixed at a constant value of 2 s^−1^ to standardize the control effort.
Input Signals: The two controller inputs are the output-voltage error and its first difference,
  (11) $$e\left[k\right]=V_{ref}\left[k\right]-V_{out}\left[k\right],\;\Delta e_{\mathrm{raw}}\left[k\right]=e\left[k\right]-e\left[k-1\right],$$
  evaluated at the controller sampling instants. The voltage-error universe was defined as e∈[−100,100] V and the first-difference universe as Δe∈[−100,100] in the scaled-derivative units defined below. The error is passed to the controller without additional normalization, $G_{e}=1$, whereas the first difference is scaled by $G_{\Delta e}=f_{sw}=225,000$ s^−1^, so that
  (12) $$\Delta e\left[k\right]=G_{\Delta e}\;\Delta e_{\mathrm{raw}}\left[k\right]=f_{sw}T_{c}\frac{de}{dt}=\frac{1}{50}\frac{de}{dt},$$
  that is, the second input is the error derivative in V/s scaled by 1/50. Because $e=V_{ref}-V_{out}$, a rising output during start-up gives a negative error derivative: an output ramp of roughly 113 V/s corresponds to de/dt≈−113 V/s and hence Δe≈−2.3, which activates the negative-big set with a membership of about 0.4, whereas steady-state operation maps into the zero set. Together with a positive-big error this combination selects the zero output rule, so the duty ramp rate is progressively reduced as the output approaches the reference; the fixed partition of Equation (15) is dimensioned to place this regulating action in the range of output slew rates that the converter actually exhibits. The scaled derivative is limited to $\pm 10^{4}$ before fuzzification, and inputs beyond the tabulated universes are handled by the linear extrapolation of the look-up table.
Control Law: The defuzzified output Δu is not applied as a direct duty command but as a rate command: it is scaled by the output gain $G_{du}$ and integrated to form the duty cycle, so that
  (13) $$\frac{dD}{dt}=K\;G_{du}\;\Delta u\left(t\right),\;D=\mathrm{sat}\;\left(\int_{0}^{t}K\;G_{du}\;\Delta u\left(\tau \right)\;d\tau ,\;0,\;0.9\right),$$
  which is realized in the model by a discrete-time integrator with forward-Euler update $D\left[k\right]=D\left[k-1\right]+K\;T_{c}\;G_{du}\;\Delta u\left[k\right]$. The integrator gain is K=1 and the output scaling gain is $G_{du}=2$ s^−1^, so that the duty-rate gain $KG_{du}=2$ s^−1^ and the nominal slew bound for |Δu|≤1 is |dD/dt|≤2 $s^{-1}$. Although the output universe is Δu∈[−1,1], centroid defuzzification of the right-shoulder positive-big set of Table 4 returns at most 2/3 when that set is fully fired, so the largest realizable duty slew rate is $KG_{du}\cdot 2/3\approx 1.33$ duty units per second. This bounded slew rate, rather than any per-step increment limit, is what produces a smooth duty trajectory instead of a bang–bang response: driving the duty cycle from zero to its nominal value of about 0.5 requires at least 375 ms even under a fully saturated fuzzy output, which is consistent with the 425 ms start-up duration reported in Section 3.4.

The integrator output is limited to D∈[0,0.9], and the limiting is applied to the integrator state itself. This clamping of the accumulated state is what provides the anti-windup action for the fuzzy loop; the integrator is initialized at zero. The commanded range is deliberately wider than the ideal steady-state operating range of 0.333–0.600 given in Table 1. For the PI controller the accumulated control command was clamped to the same interval. No explicit inductor-current limiter or zero-current blocking logic was included in the simulation model.

The output scaling gain was fixed at $G_{du}=2$ $s^{-1}$ in order to keep the FLC search space two-dimensional and thereby match the two-parameter search dimension of the PI controller. Including $G_{du}$ as an additional decision variable would have produced an asymmetric three-parameter FLC search. The physical reasoning behind the specific value $G_{du}=2\;s^{-1}$ is given at the end of this section, and the cost of holding it fixed is quantified directly by the partially joint-optimized FLC-C variant of the ablation in Section 3.6, which lowers the mean ITAE by 2.1% relative to the two-dimensional search. FLC-A, FLC-B, and FLC-C denote three different PSO-tuned FLC configurations, corresponding to membership-boundary optimization, scaling-gain optimization, and partial joint optimization, respectively.

Membership Functions: Triangular and trapezoidal membership functions are used. To keep the optimization problem low-dimensional, two scalar parameters are tuned by PSO: (i) the width of the zero region ($ZE_{width}$) and (ii) the saturation point of PB/NB ($PB_{sat}$). These two parameters define the perceptual decision boundaries of the fuzzy reasoner—the ranges over which an input is judged “negligible” versus “large”—so that the swarm search is, in effect, adapting the cognitive thresholds of the controller. Search-space boundaries are applied during the PSO execution: $ZE_{width}\in \left[0,10\right]$ and $PB_{sat}\in \left[0,50\right]$. Based on the best run, the optimized values obtained are $ZE_{width}=7.514$ V and $PB_{sat}=20.756$ V.

The PSO search was applied only to the membership-function boundaries of the voltage-error input; the change-of-error and output partitions were retained at fixed, symmetric values. This choice keeps the optimization problem two-dimensional and isolates the effect of adapting the dominant voltage-error classification boundaries. For the error input, $ZE_{width}$ is the half-width of the central zero-error region and $PB_{sat}$ is the magnitude at which the positive-big and negative-big sets reach full membership, giving

(14) $$\begin{array}{cc} {\mathrm{NB}}_{e} & =\mathrm{trap}(e;\;-100,\;-50,\;-20.756,\;0),\\ {\mathrm{ZE}}_{e} & =\mathrm{tri}(e;\;-7.514,\;0,\;7.514),\\ {\mathrm{PB}}_{e} & =\mathrm{trap}(e;\;0,\;20.756,\;50,\;100). \end{array}$$

The negative-big and positive-big sets extend to the origin so that the three sets overlap over the central region and no gap is left around $\pm ZE_{width}$. The two outer sets are finite trapezoids that return to zero at |e|=100 V, i.e., at the edge of the declared universe; in the operating range explored here the error never exceeds the 48 V start-up value, for which the positive-big set is fully active. Only the two optimized quantities $ZE_{width}$ and $PB_{sat}$ enter this partition; the change-of-error and output partitions are specified by fixed numerical parameters and are not affected by the search. The change-of-error partition was held fixed at

(15) $$\begin{array}{cc} {\mathrm{NB}}_{\Delta e} & =\mathrm{trap}(\Delta e;\;-100,\;-50,\;-5,\;-0.5),\\ {\mathrm{ZE}}_{\Delta e} & =\mathrm{tri}(\Delta e;\;-2,\;0,\;2),\\ {\mathrm{PB}}_{\Delta e} & =\mathrm{trap}(\Delta e;\;0.5,\;5,\;50,\;100). \end{array}$$

This partition is deliberately narrower around the origin than the error partition, so that a small but non-zero rate of change is still classified as approximately zero while a sustained trend is resolved into the negative-big or positive-big sets.

**Figure 4:**
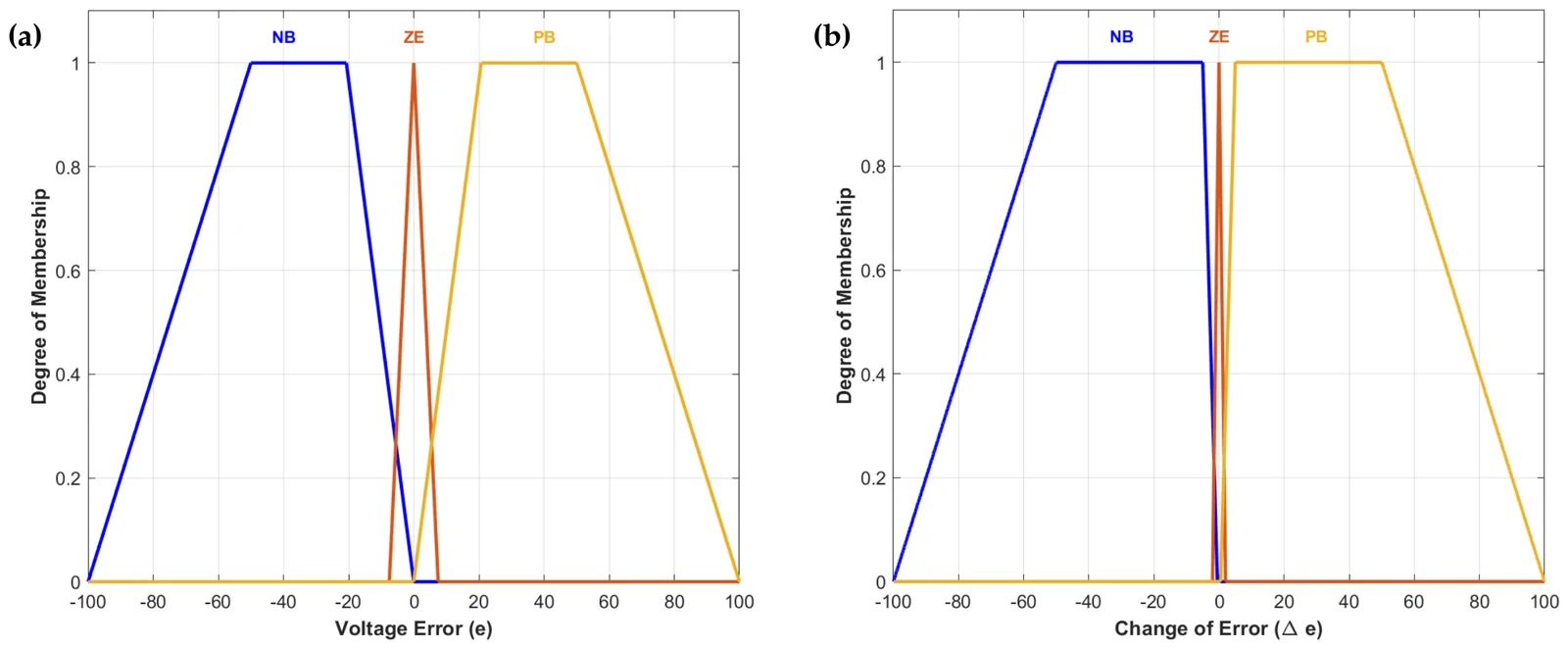
Input membership functions of the fuzzy logic controller. (**a**) Input 1: voltage error (*e*), whose boundaries are set by the offline-optimized PSO parameters $ZE_{width}=7.514$ V and $PB_{sat}=20.756$ V; (**b**) Input 2: change of error (Δe), which was held fixed and was not optimized. The output partition is listed in Table 4.

Output Membership Functions: The FLC output represents a normalized duty-cycle rate command defined over the universe Δu∈[−1,1]. Three fixed output membership functions were used, as listed in Table 4. Mamdani min–max inference and centroid defuzzification are applied to this partition to obtain the crisp normalized increment Δu[k], which is then scaled by $G_{du}$ in Equation (13).
Rule Base: A compact 3×3 rule base is used as summarized in Table 5. The rules encode the same monotonic, common-sense reasoning a human expert would apply—larger and worsening errors call for stronger corrective action—thereby realizing a transparent, cognition-like mapping from perception to control action.
Optimized Membership Functions: As illustrated in Figure 4, the PSO framework directly tunes the geometry-defining limits of the FLC membership functions. In biomimetic terms, the swarm search does not merely amplify the controller’s inputs; it reshapes the internal perceptual boundaries that determine how the fuzzy reasoner categorizes and responds to error, yielding an adapted decision map rather than a rescaled one.

The output scaling gain was designated as $G_{du}=2\;s^{-1}$ based on converter physical duty-cycle slew rate limitations and optimization search-space parity. Setting $G_{du}$ bounds the maximum duty-cycle rate of change to $\left|dD/dt\right|\leq 2\;s^{-1}$ under fully saturated fuzzy inference outputs (|Δu|≤1). This bounded integration rate prevents violent duty-cycle excursions while enabling full dynamic swing across the operating duty range (D∈[0.333,0.600]). Furthermore, fixing $G_{du}$ standardizes the control effort and enforces a strictly two-dimensional parameter search space ($ZE_{width},PB_{sat}$), maintaining dimensionality parity with the two-dimensional search space of the PI controller ($K_{p},K_{i}$).

### 2.3. MATLAB Implementation and Test Scenarios

#### 2.3.1. Realistic Simulation Model

To improve the hardware relevance of the simulation results, the MATLAB Simulink R2024b model was built as a detailed non-ideal switching model rather than an idealized one. Multi-fidelity modeling studies of DC–DC converters have shown that including device-level and parasitic effects materially improves agreement between simulation and measurement [38]; note, however, that the correlation reported in such studies rests on hardware-in-the-loop and prototype measurements, which are not available for the converter modeled here. Specifically, critical parasitic components and non-ideal characteristics derived from the hardware prototype design were integrated into the power stage. For the synchronous switching elements, the precise parameters of the IXTH200N10T N-Channel Power MOSFET (IXYS Corporation, Milpitas, CA, USA) were utilized ($R_{DS(on)}=5.5$ mΩ and $t_{r}+t_{f}\approx 65$ ns).

**Figure 5:**
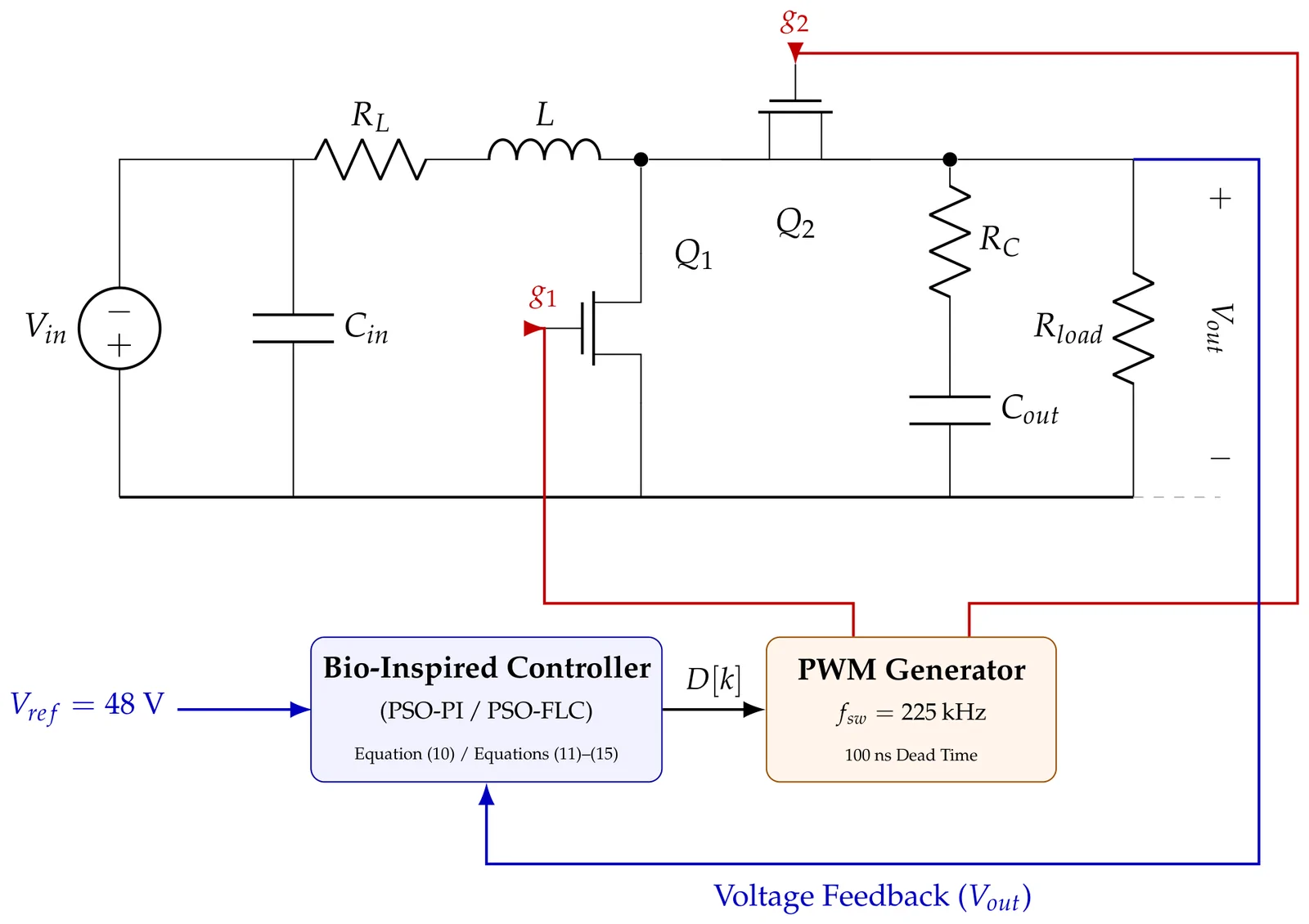
Closed-loop synchronous boost converter schematic showing the non-ideal power stage (parasitic DCR $R_{L}$, ESR $R_{C}$, MOSFET $R_{DS(on)}$), output voltage feedback sensing, and the bio-inspired controller block driving complementary synchronous MOSFET switching ($g_{1},g_{2}$) with 100 ns dead time.

Furthermore, the equivalent series resistance (ESR) of the hybrid output capacitor bank (comprising a 1 mF electrolytic capacitor and multi-layer ceramic capacitors, MLCCs) and the direct-current resistance (DCR) of the 26 μH high-current inductor were incorporated into the plant model. These were represented by lumped values of 15 mΩ and 12 mΩ, respectively. Complementary gate signals were applied with a dead time of 100 ns, and the intrinsic MOSFET body diode was retained in the switching model; the reverse-recovery charge was not parameterized as a separate loss term. Figure 5 presents the simulation schematic, in which the power stage, the controller block (interchangeable between the PI and the fuzzy look-up implementation), the gate-drive path and the output-voltage feedback are shown as separate groups.

The converter and controllers are modeled in MATLAB Simulink R2024b using the parameters in Table 1. The switching frequency is set to 225 kHz, and CCM operation is maintained across the tested operating points.

#### 2.3.2. Simulation Environment and Reproducibility

All controller comparisons were performed in MATLAB/Simulink R2024b (Update 6) using the same switch-level synchronous boost-converter model, the same electrical parameters, initial conditions, disturbance schedule and output-processing procedure. The power stage was built with Simscape and Simscape Electrical, the fuzzy inference engine with the Fuzzy Logic Toolbox, and the linear control blocks with the Control System Toolbox. The model was integrated with a fixed-step solver (automatic solver selection within the fixed-step family) at a step size of $1/(50f_{sw})=88.9$ ns, that is fifty integration steps per switching period; over the 2.5 s evaluation horizon this corresponds to approximately $2.8\times 10^{7}$ integration steps per candidate evaluation. The switching frequency was 225 kHz and the control law was executed at the solver step rate, $T_{c}=88.9$ ns (a numerical setting of the model, not a hardware execution period). At the beginning of every simulation the inductor current, the output-capacitor voltage, the PI integral state and the duty-cycle command were initialized to zero. The same initialization and numerical configuration were used for the PI and the FLC cases. Simulations were executed on a Windows 11 Pro workstation with a 16-core processor and 33.8 GB of RAM. On this machine a complete PSO run required of the order of eight hours for the FLC and about one hour for the PI controller, the difference being dominated by the cost of evaluating the fuzzy inference at every control update. The MATLAB Simulink R2024b model and the associated optimization scripts are available from the corresponding author, which permits inspection of the complete solver and block-level configuration.

Evaluating the Mamdani inference engine at every integration step proved to be the dominant computational cost of the optimization. To reduce it, the fuzzy control surface was evaluated offline on a 501×501 grid spanning e∈[−20,20] V and Δe∈[−100,100], and the resulting surface was embedded in the model as a two-dimensional look-up table using linear interpolation and linear extrapolation. Because the membership-function boundaries change with every candidate solution, the surface was regenerated from the corresponding Mamdani system for each particle evaluation; the look-up table is therefore an acceleration of the same inference, not a different controller. The grid spacing is 0.08 V on the error axis, over which the normalized output varies by less than 0.004, so the interpolation error is below 0.2% of the output range and is negligible relative to the differences between the controllers reported in Section 3. The tabulated error range is narrower than the nominal error universe because the control surface is close to saturation near $PB_{sat}=20.756$ V; errors larger than 20 V, which occur only during the start-up transient, are handled by the linear extrapolation of the table and in any case drive the duty command into the limit imposed by the integrator.

The stochastic elements of the optimization were handled as follows. Particle positions were initialized from a uniform distribution within the corresponding search bounds, and initial velocities were set to zero. Positions exceeding a search boundary were projected onto the nearest admissible value. A cost of $10^{12}$ was assigned to simulations that terminated with a non-finite signal, a solver failure, or an output voltage outside the numerical safety interval of 0–100 V. The random-seed sequence was fixed (seeds 101–110) so that every run is reproducible; because the two search spaces have different bounds, identical seeds do not imply identical initial swarms.

#### 2.3.3. Load Conditions of the Test Groups

Because the reported ripple and efficiency figures depend directly on the load, the load model applied in each test group is stated explicitly. A resistive load model was used throughout. For the input-voltage-variation tests the load resistance was fixed at $R_{load}=30\;\Omega$, corresponding to approximately 1.6 A and 76.8 W at a 48 V output. The reference-voltage-tracking tests were conducted with the same 30Ω load. For the load-transition tests the resistance was stepped from 30Ω to 15.05Ω at t=1.0 s and returned to 30Ω at t=1.5 s; these values correspond to approximately 1.6 A half-load and 3.19 A full-load operation at 48 V, the latter matching the 153 W rating in Table 1. Consequently, the input-voltage and reference-voltage test groups are half-load evaluations, whereas only the upper level of the load-step test exercises the converter at its rated power.

Three groups of large-signal tests are applied:

1. Input-voltage variation: start-up 0 → 24 V, then 24 → 28 V at t=1 s, 28 → 22 V at t=1.5 s, and 22 → 30 V at t=2 s, with $V_{ref}=48$ V throughout.
2. Load variation: the load resistance is stepped from 30 Ω to 15.05 Ω at t=1.0 s and back to 30 Ω at t=1.5 s, corresponding to a load-current step of 1.6 A to 3.19 A and back, at $V_{in}=24$ V and $V_{ref}=48$ V.
3. Reference-voltage variation: $V_{ref}$ is stepped as 0 → 48 → 55 → 45 V at $V_{in}=24$ V, with the load model as specified in Section 2.3.3.

#### 2.3.4. Definition of the Reported Efficiency

The efficiency reported in Table 6, Table 7 and Table 8 is the ratio of the averaged output power to the averaged input power,

(16) $$\eta =\frac{\bar{P_{out}}}{\bar{P_{in}}}\times 100\%=\frac{\bar{v_{out}\left(t\right)\;i_{out}\left(t\right)}}{\bar{v_{in}\left(t\right)\;i_{in}\left(t\right)}}\times 100\%,$$

where the overbar denotes averaging over an interval in steady state. For each operating point the input and output powers were averaged over the final 50 ms of the corresponding steady-state interval, and samples belonging to the transient event and to the settling interval were excluded. The loss model accounts for MOSFET conduction loss through $R_{DS(on)}$, switching-transition loss based on $t_{r}+t_{f}$, inductor copper loss through the DCR, output-capacitor loss through the equivalent ESR, and body-diode conduction during the 100 ns dead-time interval. Gate-drive power, detailed reverse-recovery charge, frequency-dependent magnetic core loss, PCB copper loss, connector loss, and the thermal dependence of component parameters were not modeled separately. The reported efficiency values should therefore be interpreted as internally consistent simulation estimates that permit comparison between the two controllers, rather than as experimentally validated absolute efficiencies.

## 3. Results

The following definitions apply to Table 6, Table 7 and Table 8. $V_{out}$ is the steady-state output voltage reached after the transient. $V_{os}$ is the peak transient deviation of the output voltage from its steady-state value, positive for an overshoot and negative for an undershoot; it is reported in volts and as a percentage of the applicable reference (48 V for Table 6 and Table 7, and the final commanded reference of each step for Table 8). $\Delta V_{out}$ is the steady-state output-voltage ripple in volts peak-to-peak (Vpp) and $\Delta i_{L}$ the steady-state peak-to-peak inductor-current ripple. $T_{st}$ is the settling time, defined as the time for the output voltage to enter and remain within a ±2% band around its final value. η is the simulated converter efficiency at the operating point, computed as in Equation (16). All values are obtained from simulation; the load condition of each test group is given in the corresponding caption and in Section 2.3.3.

### 3.1. Effect of PSO Optimization

**Figure 6:**
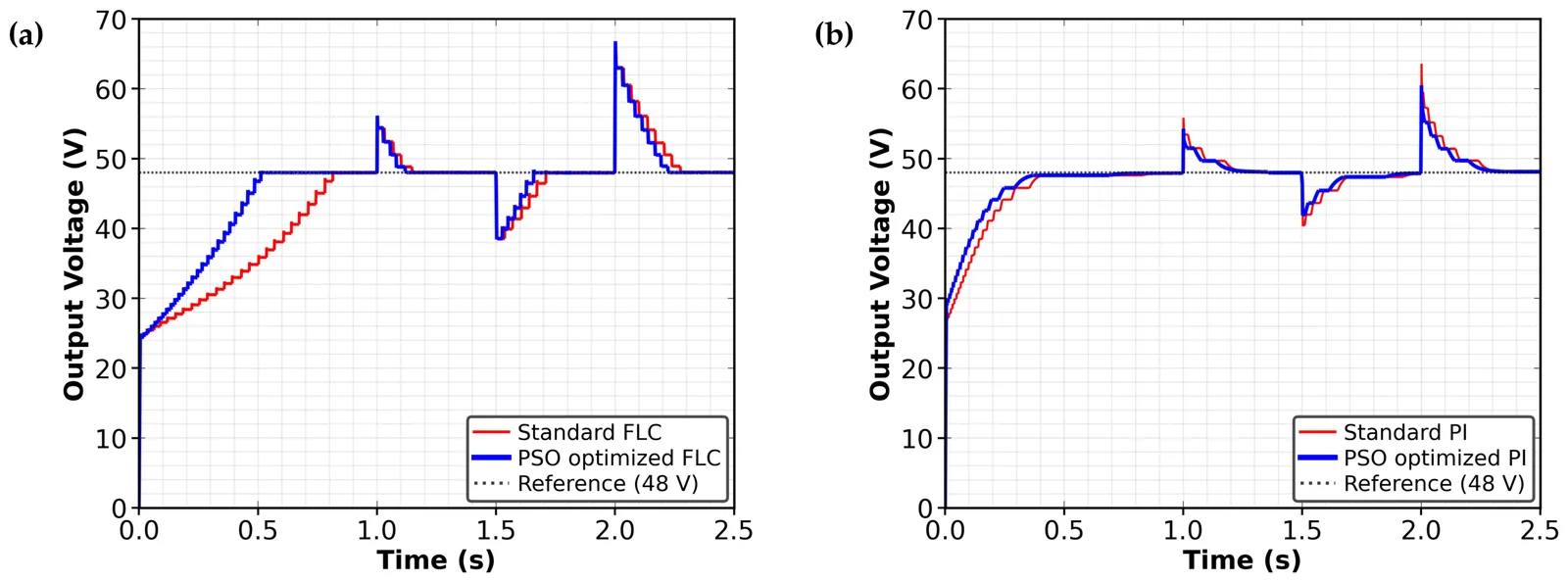
Impact of PSO-based tuning on the transient response. (**a**) Manually tuned baseline FLC versus PSO-optimized FLC; (**b**) manually tuned baseline PI versus PSO-optimized PI. The curves labelled “Standard” in the plot legends correspond to the manually tuned baseline controllers.

To quantify the impact of the optimization process, a manually tuned baseline determined by hand for stability was compared against the PSO-optimized values. For the PI controller, the baseline gains were set to $K_{p}=0.4$ and $K_{i}=15.0$, whereas the PSO algorithm converged to higher gains of $K_{p}=0.7778$ and $K_{i}=19.7972$. Similarly, for the FLC, the baseline membership parameters ($ZE_{width}=1.0$ V, $PB_{sat}=30.0$ V) were optimized to $ZE_{width}=7.514$ V and $PB_{sat}=20.756$ V. As shown in Figure 6, these optimized parameters shift the transient response of both controllers from a slow overdamped behavior to a faster settling profile. The comparison is against a manually tuned baseline chosen for stability rather than for performance, so it indicates that the search moves the controllers away from a conservative starting point; the benefit of the proposed parameterization relative to an equally optimized alternative is quantified separately by the ablation of Section 3.6.

### 3.2. Input-Voltage Variation Waveforms

**Figure 7:**
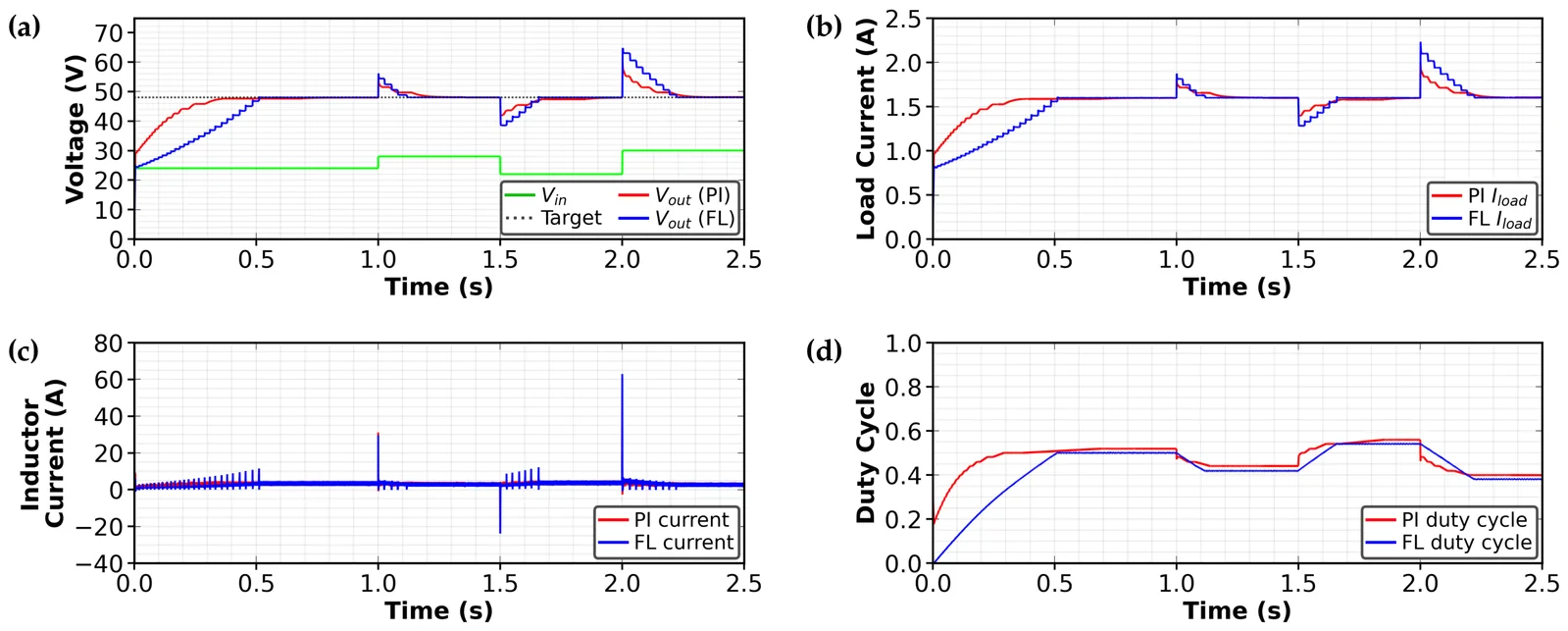
MATLAB Simulink R2024b waveforms for input-voltage variation at $V_{ref}=48$ V (red: PSO-PI, blue: PSO-FLC). (**a**) $V_{in}$ and $V_{out}$; (**b**) Load current response; (**c**) Inductor current response; (**d**) Duty-cycle evolution.

Figure 7 reports waveforms for wide input-voltage changes. For wide input variations, PSO-FLC consistently reaches steady state faster than PSO-PI. For example, in the 24 → 28 V transition, the FLC settles in 85 ms compared to 240 ms for PI. Similarly, in the 22 → 30 V transition, the FLC settles in 174 ms while PI requires 380 ms.

Panels (b) and (d) of Figure 7 show that the load current stays essentially constant through the input transitions, as expected for a regulated output into a fixed resistance, while the duty cycle tracks the new conversion ratio within the same interval as the output voltage; the duty excursion is larger and less damped for the PSO-FLC, which is the signature of the more aggressive action discussed in Section 4.

On the other hand, PSO-PI exhibits the lower peak deviation in all three input-voltage rows of Table 6. In the 22 → 30 V transition, the PI overshoot is 12.567 V while the FLC overshoot reaches 19.181 V. Furthermore, as detailed in Table 6, the input voltage level directly impacts the converter’s simulated efficiency and inductor current ripple.

### 3.3. Load Variation Waveforms

**Figure 8:**
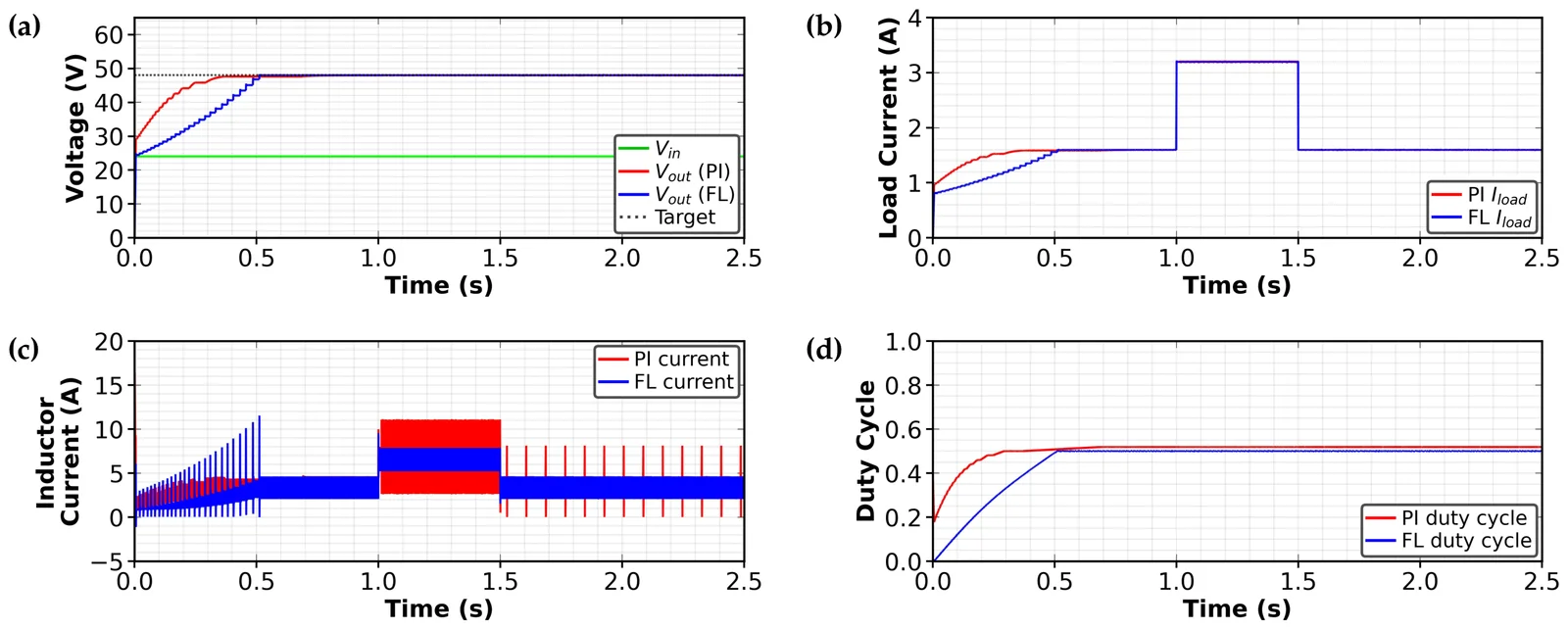
MATLAB Simulink R2024b waveforms for load variation at $V_{in}=24$ V and $V_{ref}=48$ V (red: PSO-PI, blue: PSO-FLC). (**a**) Output-voltage response; (**b**) Load current response; (**c**) Inductor current response; (**d**) Duty-cycle evolution.

Figure 8 shows the load regulation performance, and the corresponding metrics are collected in Table 7. During load transients, PSO-PI yields shorter settling times (2–4 ms) than PSO-FLC (4.5–10 ms). Both controllers regulate the output near 47.9 V with small overshoot and similar simulated efficiency.

Panel (b) of Figure 8 shows the commanded load-current step itself, and panel (d) shows that the duty cycle returns to essentially the same steady-state value after the transient, confirming that the operating point is unchanged by the disturbance. Panel (c) indicates that the inductor current tracks the new load level without a sustained offset.

Beyond settling time, the steady-state metrics under full load (3.19 A) show no comparable penalty: although the PSO-PI settles faster, the inductor-current ripple is essentially the same for the two controllers, 2.091 A for the PSO-FLC against 2.047 A for the PSO-PI.

### 3.4. Reference-Voltage Variation Waveforms

**Figure 9:**
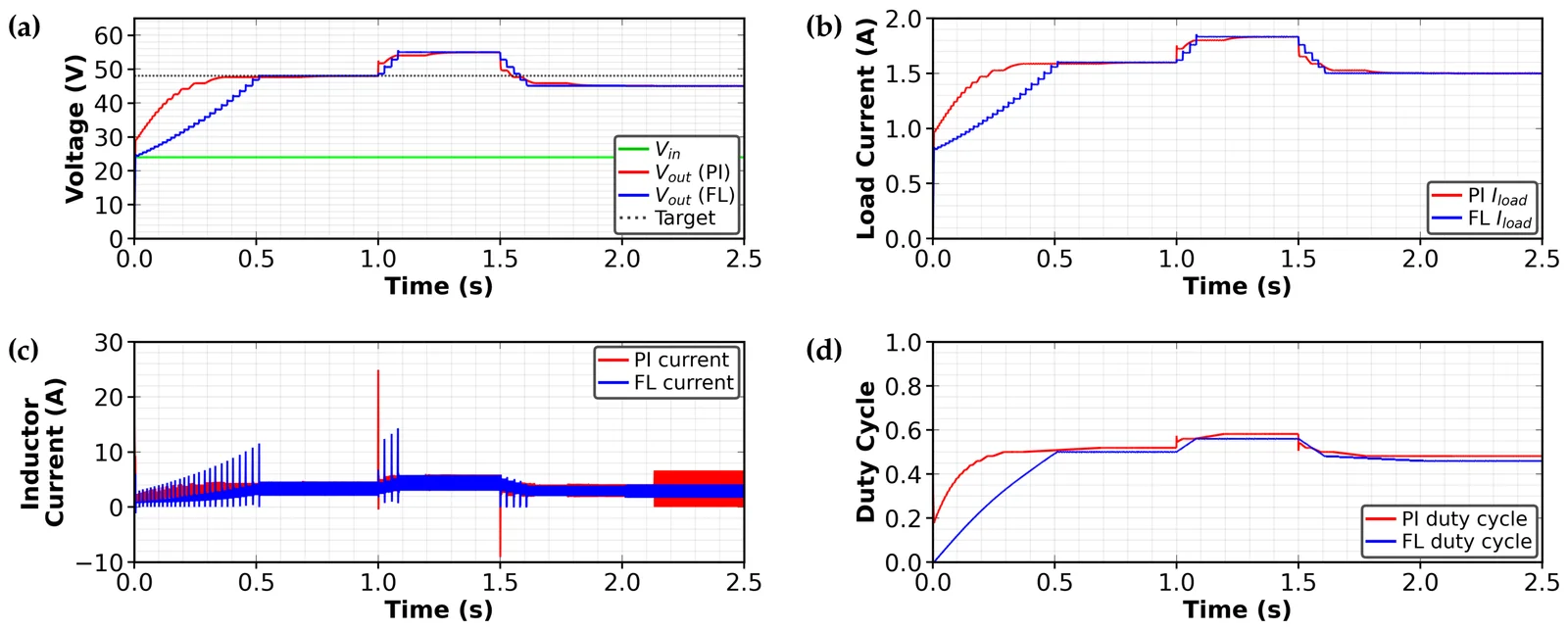
MATLAB Simulink R2024b waveforms for reference-voltage variation at $V_{in}=24$ V (red: PSO-PI, blue: PSO-FLC). (**a**) Output-voltage tracking; (**b**) Load current response; (**c**) Inductor current response; (**d**) Duty-cycle evolution.

Figure 9 evaluates tracking performance for the reference sequence 0 → 48 → 55 → 45 V. For reference tracking, PSO-FLC demonstrates faster settling for both upward and downward steps. The 48 → 55 V step settles in 128 ms (FLC) versus 240 ms (PI). The 55 → 45 V step settles in 90 ms (FLC) versus 305 ms (PI).

The efficiency metrics in Table 8 vary only marginally over the 45–48 V references (93.55–93.64%), whereas the 55 V reference shows a markedly higher figure (about 95.3%). Panels (b)–(d) of Figure 9 show the corresponding load-current, inductor-current and duty-cycle responses: because the load is resistive, the current follows each reference level proportionally, and the duty cycle settles at the value dictated by the new conversion ratio. With the constant 30 Ω load, the nominal output powers are 67.5 W at 45 V (1.50 A), 76.8 W at 48 V (1.60 A) and approximately 100.8 W at 55 V (1.83 A). The higher simulated efficiency at the 55 V reference therefore coincides with an increase in transferred output power, which reduces the relative contribution of the approximately fixed loss terms; it should not be interpreted as evidence that increasing the output voltage intrinsically improves converter efficiency.

### 3.5. Summary of Performance Metrics

The performance metrics extracted from the MATLAB Simulink R2024b simulations are summarized in Table 6, Table 7 and Table 8, organized by test scenario for readability. Collectively, they highlight an application-dependent trade-off: PSO-FLC provides the shorter settling time in every large-signal input-voltage and reference-voltage transition (Table 6 and Table 8), whereas PSO-PI provides the smaller peak voltage deviation in every input-voltage transition (Table 6). The ordering reverses for load steps at the nominal input (Table 7), where PSO-PI settles in 2–4 ms against 4.5–10 ms for PSO-FLC, while steady-state regulation and efficiency remain comparable.

To provide an overarching side-by-side synthesis of the two bio-inspired control strategies, Table 9 consolidates the key dynamic performance metrics across all evaluated input-voltage, load, and reference-tracking scenarios. This summary explicitly highlights the core engineering trade-off: PSO-FLC delivers superior large-signal adaptation speed (up to 70.5% reduction in settling time), whereas PSO-PI provides tighter peak transient voltage containment during line disturbances.

### 3.6. Ablation: Boundary Tuning Versus Scaling-Gain Tuning

The comparison of Section 3.2, Section 3.3, Section 3.4 and Section 3.5 establishes how the boundary-tuned FLC behaves relative to a PSO-tuned PI controller, but it does not by itself identify which part of the fuzzy parameterization produces that behavior. To isolate that contribution, three FLC variants were optimized under an identical budget—20 particles, 100 iterations, ten independent runs, and the same ITAE objective, disturbance profile and seed sequence used throughout this study:

- FLC-A, the proposed variant, searching the two membership-boundary parameters ($ZE_{width}$, $PB_{sat}$) with the scaling gains held fixed;
- FLC-B, representing the conventional practice reported in the literature [31,32], searching the input scaling gains ($G_{e}$, $G_{\Delta e}$) with $G_{du}=2\;s^{-1}$ and the membership geometry held at the manually tuned baseline values $ZE_{width}=1.0$ V and $PB_{sat}=30.0$ V of Section 3.1;
- FLC-C, a three-parameter partial joint search over ($ZE_{width}$, $PB_{sat}$, $G_{du}$), which raises the search-space dimension from two to three.

FLC-C is a partial rather than a fully joint optimization of the fuzzy controller, and is referred to as such throughout: the input scaling gains $G_{e}$ and $G_{\Delta e}$, the change-of-error and output partitions of Equation (15) and Table 4, and the 3×3 rule base of Table 5 are held fixed in all three variants. A fully joint optimization would place those quantities in the search space as well, which lies outside the scope of the present ablation.

FLC-A and FLC-B therefore have the same number of free parameters and differ only in which quantities the swarm is allowed to move. Table 10 reports the attained cost and the two large-signal input-voltage transitions for all four controllers, together with the search bounds enforced during the PSO execution and the parameter vector of the best run of each variant. The bounds imposed on $G_{e}$, $G_{\Delta e}$ and $G_{du}$ each bracket the value that FLC-A holds fixed, so neither FLC-B nor FLC-C was prevented by its bounds from reproducing the FLC-A configuration; the differences in attained cost reported below are therefore differences in what the swarm found within a permissive box and not artefacts of an exclusionary one.

In Table 10, $V_{os}$ percentages are referred to the 48 V nominal bus voltage, as in Table 6. FLC-B holds the membership geometry at the manually tuned baseline of Section 3.1 ($ZE_{width}=1.0$ V, $PB_{sat}=30.0$ V); FLC-A and FLC-C hold $G_{e}=1$ and $G_{\Delta e}=f_{sw}$. Search bounds and best parameter vectors are listed componentwise in the same order as the tuned parameters of the row above; the $G_{\Delta e}$ interval $\left[0.1,5\right]\;f_{sw}$ corresponds to $\left[2.25\times 10^{4},1.125\times 10^{6}\right]$ $s^{-1}$. Every bound listed for FLC-B and FLC-C brackets the value that FLC-A holds fixed ($G_{e}=1$, $G_{\Delta e}=f_{sw}$, $G_{du}=2$ $s^{-1}$), so both searches were free to recover the FLC-A configuration. Best parameter vectors are those of the run attaining the lowest final ITAE cost of the ten. FLC-C is a three-parameter partial joint optimization and not a fully joint structural search of the fuzzy controller.

Three observations follow. First, boundary tuning outperforms scaling-gain tuning both on the criterion the search actually minimizes and on settling time. FLC-A attains a mean ITAE of 2.9674 against 3.3850 for FLC-B, a reduction of 12.3%, and settles in 85.0 ms against 142.0 ms on the 24→28 V step and 118.0 ms against 195.0 ms on the 28→22 V step, reductions of 40.1% and 39.5%. Because the two variants are given the same optimizer, the same budget and the same number of free parameters, and differ only in which quantities the swarm may move, this is a controlled attribution: relocating the internal decision boundaries is a more effective use of two degrees of freedom than rescaling the controller inputs. The separation is also large relative to the variability of the search itself: the difference between the two mean costs, 0.418, is roughly nine times the run-to-run standard deviation of either variant (0.0425 and 0.0512), so it is large relative to the run-to-run dispersion observed for the two searches. The margin is reported in this dispersion-referenced form rather than as a paired significance test: as noted in Section 2.3.2, the two variants search spaces with different bounds, so a common seed sequence does not produce a common initial swarm and the ten runs of FLC-A and FLC-B are not paired realizations of a single random draw. Both fuzzy variants in turn attain a lower ITAE than the PSO-PI benchmark, FLC-B by 13.1% and FLC-A by 23.8%.

Second, the advantage is not uniform across metrics. FLC-B produces the smaller peak deviation on both transitions, 14.3% against 17.0% and −16.5% against −20.0%. The pattern reproduces the PSO-PI/PSO-FLC trade-off of Section 3.5 and has the same origin: the unweighted ITAE criterion of Equation (8) rewards rapid error reduction and places no penalty on peak excursion, so the additional agility that boundary tuning provides is expressed as a larger initial deviation. Boundary tuning therefore does not remove the speed–overshoot trade-off identified in Section 4.2; it moves the closed loop further along it. Applications with narrow voltage headroom would need the composite objective discussed in Section 4.2 rather than a different parameterization.

Third, extending the search to include the output gain produces a small further improvement rather than a qualitative change. FLC-C lowers the mean ITAE by 2.1% relative to FLC-A (2.9050 against 2.9674), shortens the 24→28 V settling time by 4 ms, and also reduces the peak deviation slightly, from 17.0% to 16.3%. The gain is consistent in sign across all three metrics but modest for a 50% increase in search-space dimension: the difference of 0.062 in mean cost is only about one and a half times the run-to-run standard deviation of either variant, in contrast to the roughly nine-fold separation between FLC-A and FLC-B. The two-dimensional variant also preserves the dimensional parity with the two-parameter PI baseline that motivated fixing $G_{du}$ in Section 2.2.3. Read as a best-against-best comparison rather than a dimension-matched one, the strongest fuzzy configuration identified in this study is FLC-C, whose mean ITAE of 2.9050 lies 25.4% below the 3.8961 of the PSO-PI benchmark, against the 23.8% margin of the two-parameter FLC-A. Releasing the third degree of freedom therefore widens rather than narrows the advantage of the fuzzy controller over the linear baseline, and both readings are reported here. The FLC-A comparison of Section 3.2, Section 3.3, Section 3.4 and Section 3.5 is the conservative one: with an equal number of free parameters, the margin it reports cannot be attributed to the fuzzy controller having been granted a larger search space than the baseline. FLC-C bounds what the same parameterization attains under this budget once that restriction is lifted. FLC-A is retained as the configuration reported in detail for the former reason, and the two-parameter and three-parameter results are given side by side in Table 10 so that neither reading is withheld.

Taken together, the ablation supports the central methodological proposition of this work—that the membership-function boundaries are a more productive target for the swarm search than the external scaling gains—while locating the benefit specifically in attained cost and large-signal speed rather than in transient voltage containment.

### 3.7. Sensitivity to the Control Update Rate

The comparison reported in Section 3.2, Section 3.3, Section 3.4 and Section 3.5 was obtained with the control law evaluated at the solver step ($T_{c}=88.9$ ns, fifty samples per switching period), which is a numerical setting of the model rather than a hardware execution period. Because the control update rate, the computation delay and the finite resolution of a physical digital implementation are all known to alter the closed-loop dynamics relative to a continuous-time realization [39,40,41], the optimized parameter sets were re-evaluated at a realizable update rate before any implementation-oriented conclusion is drawn.

For this evaluation the controller block was preceded by a zero-order hold and the control law was executed once per switching period, $T_{c}=T_{sw}=4.44\;\mu$s (225 kHz update rate), with the membership boundaries, PI gains and disturbance profile held at the values used throughout Section 3. One scaling quantity cannot be held constant in this test. The second fuzzy input is formed as $\Delta e\left[k\right]=G_{\Delta e}\;T_{c}\;de/dt$ in Equation (12), so the effective derivative gain seen by the fuzzy reasoner is set by the product $G_{\Delta e}T_{c}$ and not by $G_{\Delta e}$ alone. Leaving $G_{\Delta e}=f_{sw}$ unchanged while increasing $T_{c}$ by a factor of fifty would therefore magnify the derivative input by the same factor and drive the fixed change-of-error partition of Equation (15) into permanent saturation, which would represent a different controller rather than the same controller at a lower update rate. The first-difference scaling was consequently re-derived for the new update rate as $G_{\Delta e}=0.02/T_{c}=4500\;s^{-1}$, so that the product $G_{\Delta e}T_{c}=0.02$ defining the derivative input is identical to that of the solver-step model and the comparison isolates the effect of the update rate alone. This substitution is a deterministic rescaling of a single normalization constant and not an additional tuning step: $G_{\Delta e}$ is obtained in closed form from the new $T_{c}$ by the identity $G_{\Delta e}T_{c}=0.02$, no search was carried out over it, and the PSO was not re-run for this evaluation. The membership boundaries, the PI gains, the objective, the disturbance profile and every other parameter were left exactly as optimized at the solver step, so the controller evaluated at $T_{c}=4.44\;\mu$s is the same controller as in Section 3.2, Section 3.3, Section 3.4 and Section 3.5, executed at a lower update rate. Any residual change in performance is therefore attributable to the update rate and the hold, and not to a re-optimized controller. By holding the product $G_{\Delta e}T_{c}$ invariant, the change-of-error signal supplied to the fuzzy inference engine remains functionally identical to that of the solver-step model across all operating conditions, so the margin between the derivative input and the saturation threshold of its partition is preserved throughout. For the start-up ramp quantified in Section 2.2.3 (de/dt≈−113 V/s) the input reaches Δe[k]≈−2.3, which places the operating point on the active, graded slope of the negative-big set of Equation (15) at a membership of $\mu_{NB}\approx 0.40$, well short of the Δe=−5 breakpoint at which that set reaches full membership. Had $G_{\Delta e}$ been left unscaled at $f_{sw}$, the same ramp would have produced Δe[k]≈−113, exceeding the saturation breakpoint more than twenty-fold and driving the set into complete saturation. Keeping Δe inside its unsaturated, graded range preserves the fine input sensitivity of the rule base and underpins the stable inference behavior observed during the transients of Section 3.

Introducing the sampling delay and the hold lag produced a bounded degradation for both controllers. Table 11 reports the absolute settling times and peak deviations at both update intervals for the three large-signal input-voltage transitions. Over those six cases the peak overshoot increased by 3.0–5.3% and the settling time by 4.5–8.0% relative to the solver-step case. In this table, $V_{os}$ percentages are referred to the 48 V nominal bus voltage, as in Table 6. The switching-period columns were obtained with the membership boundaries, the PI gains, the objective and the disturbance profile held exactly as optimized at the solver step; the only quantity changed is the first-difference normalization $G_{\Delta e}$, which is rescaled deterministically to $0.02/T_{c}=4500$ $s^{-1}$ so that the product $G_{\Delta e}T_{c}$ defining the derivative input is unchanged. No parameter was re-optimized and the PSO was not re-run. Closed-loop stability was maintained in every scenario, and the ordering of the two controllers was unchanged: the PSO-FLC retained the shorter settling time in the large-signal input-voltage and reference transitions, and the PSO-PI retained the smaller peak deviation. The absolute settling times reported in Table 6, Table 7 and Table 8 should therefore be read as optimistic with respect to a digital realization, whereas the comparative conclusions of Section 3.5 are robust to a realizable control update rate.

### 3.8. Behavior Under an Explicit Inductor-Current Limit

Every result reported so far was obtained without any current-limiting mechanism, and Section 4.3 quantifies the transient excursions that follow from that choice. Because a converter built to these specifications would carry cycle-by-cycle protection, the two tuned controllers were re-evaluated with such a limiter added to the switch-level model, so that the consequences of the unconstrained optimization can be assessed rather than only stated.

The limiter compares the instantaneous inductor current against a threshold $I_{lim}=10$ A, placed at the saturation rating of the selected inductor. When the threshold is reached the gate command of the low-side switch is withdrawn for the remainder of the switching period, and a separate zero-crossing comparator blocks the synchronous rectifier so that the inductor current cannot reverse. Two properties of this test must be stated explicitly. First, neither controller was re-tuned: the membership boundaries, the PI gains, the objective, the scaling gains and the disturbance profile are exactly those used throughout Section 3, and no PSO run was repeated. The evaluation therefore measures how the already-optimized control laws behave inside a protected converter; it is not a re-optimization under a current constraint, which remains future work as set out in Section 4.6. Second, the limiter is inactive in steady state at every operating point tested: the peak of the steady-state ripple stays near 5 A against the 10 A threshold, so the ripple and efficiency figures of Table 6, Table 7 and Table 8 are unaffected by its presence.

**Figure 10:**
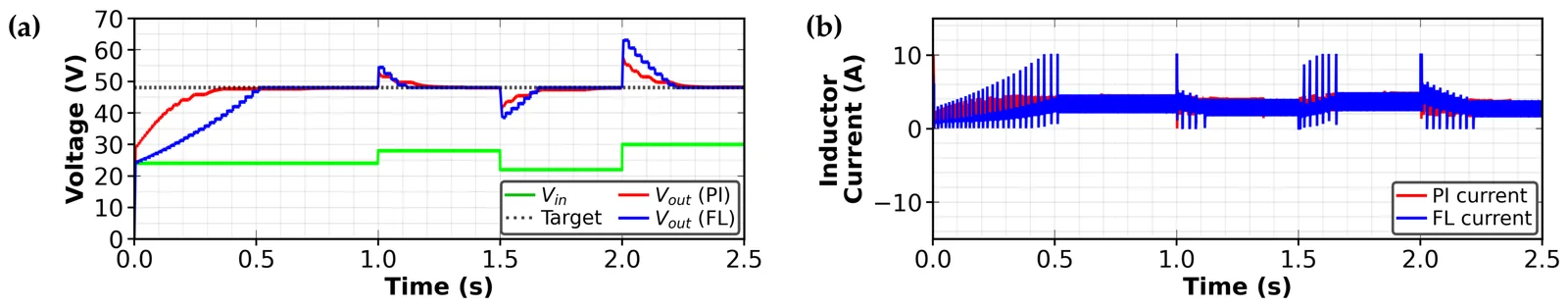
MATLAB Simulink R2024b waveforms for input-voltage variation at $V_{ref}=48$ V with the cycle-by-cycle inductor-current limit ($I_{lim}=10$ A) and reverse-current blocking active (red: PSO-PI, blue: PSO-FLC). (**a**) $V_{in}$ and $V_{out}$; (**b**) inductor current, confined to the −0.20 A to 10.21 A band by the limiter. The controllers are those optimized in Section 3 and were not re-tuned for this evaluation.

Figure 10 shows the resulting output-voltage and inductor-current waveforms for the input-voltage profile, and Table 12 collects the corresponding metrics; the unconstrained counterparts are those of Table 6 and Section 4.3. In Table 12, $V_{os}$ percentages are referred to the 48 V nominal bus voltage, as in Table 6.

The forward threshold is reached on the start-up ramp and on the 24→28 V and 22→30 V transitions for both controllers, and on the 28→22 V transition for the PSO-FLC only; the PSO-PI peaks at 5.13 A there and its forward current is therefore never clamped. Reverse-current blocking keeps $i_{L,min}$ at or above −0.20 A throughout, against the −23.31 A and −19.65 A excursions of the unconstrained PSO-FLC and PSO-PI reported in Section 4.3. The unconstrained counterparts of the settling times listed here are 240.0, 400.0 and 380.0 ms for the PSO-PI and 85.0, 118.0 and 174.0 ms for the PSO-FLC (Table 6).

Four observations follow. First, the transient current stress is removed. The inductor current is confined to the band from −0.20 A to 10.21 A over the whole record for both controllers, against the 62.42 A forward and −23.31 A reverse excursions of the unconstrained PSO-FLC and the 57.94 A forward and −19.65 A reverse excursions of the unconstrained PSO-PI. The clamped peaks lie between 10.15 and 10.21 A and no minimum falls below −0.20 A, so each protection threshold is overshot by at most 0.21 A. Both residuals are of the same size because both have the same origin: the current that accumulates during the single switching period the comparator and the gate logic need to act. They set the practical accuracy of a protection scheme of this kind. The excursions that Section 4.3 identifies as the principal obstacle to hardware transfer are therefore eliminated by a protection scheme of the kind any practical implementation would carry.

Second, the closed loop remains stable while the limit is active. Panel (a) of Figure 10 shows that the output recovers to the 48 V reference after every transition, with no sustained oscillation, no loss of regulation and no hunting between the limited and unlimited modes. During start-up the limiter is continuously active for both controllers and the output rises in the stepped, charge-limited manner characteristic of a current-limited converter, which also bounds the inrush current at the same ceiling.

Third, the limiter reduces rather than aggravates the peak voltage deviation on the two upward transitions. On the 22→30 V step, the event responsible for the largest excursions in Table 6, the overshoot falls from 19.181 V to 15.28 V for the PSO-FLC and from 12.567 V to 9.62 V for the PSO-PI; on the 24→28 V step it falls from 8.141 V to 6.78 V and from 6.030 V to 4.94 V respectively. The four reductions lie between 16.7% and 23.5%. The mechanism is direct: the same clamp that caps the inductor current also caps the excess charge delivered to the output capacitor during the interval in which the duty command is still too large. The 28→22 V transition behaves differently, and the difference is instructive. There the output undershoots because energy is momentarily unavailable rather than in excess, so the depth of the excursion is set at the instant of the step and a current ceiling cannot reduce it—indeed the PSO-PI never reaches the threshold there at all, peaking at 5.13 A: the undershoot moves from −9.613 V to −9.56 V for the PSO-FLC and from −6.220 V to −6.17 V for the PSO-PI, changes of under one per cent. What the clamp does affect on that transition is the recovery, which is slower because the current available to restore the output is now bounded—a separation of the two effects that is visible again in the settling times discussed next.

Fourth, and most consequential for the comparison reported in this paper, the ordering of the two controllers is preserved. With the limiter active the PSO-FLC still settles faster than the PSO-PI on all three input-voltage transitions, by 66.2%, 61.1% and 32.9% against 64.6%, 70.5% and 54.2% in the unconstrained case. The absolute times move in both directions. Four of the six lengthen, by 26.4% to 47.9%, because the clamp withholds current during precisely the interval in which the controller is restoring the output. One is unchanged: the PSO-PI settles the 28→22 V step in 401 ms against 400 ms without the limiter, and since its forward current is never clamped on that transition, this case isolates the effect of reverse-current blocking alone and shows it to be negligible for the recovery dynamics. One shortens: the PSO-PI settles the 22→30 V step 13.7% faster with the limiter than without it, because that transition produces its largest unconstrained overshoot and capping the charge delivered to the output capacitor removes more settling time than the current restriction adds. The comparative conclusion of Section 3.5 therefore survives the introduction of a protection scheme, with a narrower but uniformly retained margin.

The unconstrained results of Section 3.2, Section 3.3, Section 3.4 and Section 3.5 are retained as the primary comparison because they characterize the two tuned control laws themselves, free of any interaction with a protection scheme whose threshold and implementation are design choices rather than properties of the controllers. The present section establishes what that comparison implies once such a scheme is present.

## 4. Discussion

### 4.1. Positioning Relative to Related Studies

The literature surveyed in Section 1 applies swarm intelligence to a wide range of converter topologies, but a directly comparable benchmark for the present study is not available. Reported results differ in topology (buck, buck–boost, interleaved, dual active bridge), in power and voltage level, in the magnitude and type of the applied disturbance, and in whether the figure quoted is a rise time or a settling time. A numerical side-by-side table across such heterogeneous conditions would suggest a controlled comparison that has not in fact been performed, and is therefore deliberately not presented here.

What can be stated is qualitative. Many of the PSO-based converter studies reviewed restrict the search to controller gains or to the input/output scaling factors of a fuzzy controller; the contribution of the present work is the direct structural parameterization evaluated here, in which the membership-function boundaries themselves are placed inside the search space. Relative to that body of work, the results reported in Table 6, Table 7 and Table 8 indicate that this parameterization yields a closed loop capable of fast large-signal recovery, at the cost of a larger peak excursion under the most severe input transition. Whether that trade-off is favourable relative to scaling-gain-only tuning is addressed directly by the ablation of Section 3.6, in which a scaling-gain-only variant was implemented on the identical 153 W synchronous boost plant and disturbance profile and optimized under the same budget. A comparable benchmark against other metaheuristics or against established non-linear control designs would require re-implementing those approaches on the same plant, which is left to future work.

### 4.2. Analysis of Controller Performance Trade-Offs

The simulation results indicate that no single controller dominates in all metrics. When the operating point changes substantially (large input or reference steps), the PSO-FLC can apply more aggressive nonlinear action, resulting in shorter settling times. However, aggressive action may also increase overshoot for certain disturbances.

A notable observation from Table 6 is the significant voltage overshoot ($V_{os}=19.181$ V, corresponding to 40.0%) exhibited by the PSO-FLC during the extreme 22→30 V input voltage transition. Because both controllers were optimized under the exact same unweighted ITAE objective (Equation (8)), the cost function prioritizes rapid error reduction via the time-multiplier term t·|e(t)| rather than penalizing initial peak excursions. Consequently, the swarm search drives the fuzzy boundaries toward an aggressive nonlinear control surface near $PB_{sat}=20.756$ V to minimize long-term settling time, accepting a higher initial peak deviation as a mathematical trade-off. While a composite objective carrying explicit peak-voltage and peak-current penalties (Equation (17)) could constrain this overshoot, maintaining the single ITAE criterion was intentional in this study to evaluate both controllers under a standard benchmark. In applications with strict voltage headroom the appropriate remedy is the composite objective rather than a larger search space: the ablation of Section 3.6 shows that releasing $G_{du}$ into a joint three-dimensional search reduces the peak deviation on the two large-signal steps only from 17.0% to 16.3% and from −20.0% to −19.2%, whereas the excursion is set primarily by the absence of a peak-error term in the criterion. In the studied converter, load steps at the nominal input are handled well by both controllers. The PI solution shows faster settling in the provided load-step tests, whereas the fuzzy controller exhibits comparable steady-state regulation and efficiency.

From a design perspective:

- If overshoot constraints are strict, PSO-PI is the less demanding of the two, since its worst-case excursion on the 22 → 30 V step reaches 60.7 V against 67.2 V for the PSO-FLC. It must be emphasized, however, that neither controller as tuned here remains within a standard 60 V/63 V component rating on that step; deploying either one at this input range would require higher-rated devices, an explicit derating margin, or an overshoot-constrained re-tuning of the objective function.
- If the application prioritizes rapid adaptation across wide operating ranges and reference tracking, PSO-FLC offers a strong advantage, particularly when adequate transient voltage margin is available at the system level.

### 4.3. Inductor-Current Excursions During Large-Signal Transients

Panels (c) of Figure 7 and Figure 9 show that the inductor current departs substantially from its steady-state envelope at the instants when the input voltage or the reference is stepped, with both large positive peaks and, for some transitions, brief negative excursions. The two step families produce these peaks by different mechanisms. For an upward reference step the commanded output rises, the controller extends the on-time and the inductor is charged rapidly. For an upward input-voltage step the required duty ratio instead falls—from D=0.542 at $V_{in}=22$ V to D=0.375 at $V_{in}=30$ V for the same 48 V output—so immediately after the step the duty is still at its previous, larger value while the on-interval slope $V_{in}/L$ has risen from 0.85 to 1.15 A/μs. The volt–second balance is therefore violated for as long as the controller needs to reduce the duty, the inductor current builds up cycle by cycle, and the excess charge is delivered to the output capacitor, which is why the same event also produces the largest voltage overshoot in Table 6. How large the excursion becomes depends on how quickly the controller withdraws the duty command, so the magnitude of the excursion differs markedly between the two controllers on the same transition, as the values reported below show. The negative excursions follow from the recovery: as the output overshoots, the duty command is driven towards its lower limit, the inductor then sees $(V_{in}-V_{out})/L\approx -1.43$ A/μs at the 67.2 V peak, and because synchronous rectification allows bidirectional current flow the current is not clamped at zero as it would be in a diode-rectified stage.

These excursions are a design-relevant consequence of the aggressive large-signal action that gives the PSO-FLC its short settling times, and they are not captured by any of the metrics in Table 6, Table 7 and Table 8, whose only current metric is the steady-state ripple. The analytical component-sizing calculation gives a nominal design peak current of approximately 8.21 A at full load, and the selected inductor was specified with a saturation-current rating above 10 A. The unconstrained switch-level simulations in Figure 7c, Figure 8c and Figure 9c exhibit brief positive and negative current excursions outside this steady-state design envelope. As noted in Section 2.2.3, no active inductor-current limiter or zero-current blocking mechanism was included in the simulation model. These excursions are therefore to be interpreted as unconstrained model-predicted transients and not as hardware-safe operating points. A practical implementation would require a cycle-by-cycle current limit near the inductor rating, synchronous-rectifier reverse-current blocking, and re-optimization of the controller under the resulting current constraint. Such a re-optimization would require the constraint to be carried by the objective itself, for example as the composite criterion

(17) $$J=J_{ITAE}+w_{v}\;V_{os}+w_{i}\max\;\left(0,\;\max_{t}i_{L}\left(t\right)-I_{lim}\right),$$

in which $I_{lim}$ is the protection threshold and the weights $w_{v}$ and $w_{i}$ price a peak voltage excursion and a violation of the current limit against accumulated tracking error. The unweighted criterion of Equation (8) used throughout this study contains neither term, which is precisely why the searches reported above are free to purchase settling time with voltage and current stress. The steady-state ripple values in Table 6, Table 7 and Table 8 do not describe these short-duration current stresses.

Implementing active current-constrained control strategies [42] and accounting for inductor core saturation alongside thermal derating effects [43] represent essential engineering measures for safe hardware deployment.

Quantitative evaluation of the inductor-current waveforms in Figure 7c shows that both controllers reach their transient current maxima on the 22→30 V input transition and that the two magnitudes are of the same order. For the PSO-FLC the current reaches 62.42 A on that transition, while the 28→22 V transition produces a reverse-current excursion of −23.31 A caused by unblocked synchronous rectification. The PSO-PI reaches 57.94 A on the same 22→30 V transition and 30.67 A on the 24→28 V transition, and the 28→22 V transition drives it to a reverse excursion of −19.65 A through the same unblocked reverse conduction. In the load-step and reference-tracking scenarios of Figure 8c and Figure 9c the ordering is reversed, the PSO-PI drawing the larger peaks (11.04 A and 24.5 A against 9.56 A and 13.9 A for the PSO-FLC). Every one of these peaks exceeds the 8.21 A nominal full-load design peak, and all of them except the 9.56 A load-step peak of the PSO-FLC also exceed the saturation rating of the selected inductor; the worst case of the PSO-FLC exceeds the nominal design peak by almost an order of magnitude. The two controllers are therefore exposed to transient current stress of the same order on the input-voltage transitions: 62.42 A against 57.94 A on the worst step, a difference of 7.7%, against a factor of about seven by which both exceed the 8.21 A design peak. The large excursions are consequently attributable to the objective rather than to the controller structure—a time-weighted error criterion that contains no current term rewards rapid error reduction and prices neither voltage nor current excursion, and it does so identically for the linear and the fuzzy parameterization. The shorter settling time of the PSO-FLC is therefore not purchased with a current penalty relative to the PSO-PI; both tuned control laws leave the steady-state design envelope by a wide margin, and the remedy lies in the objective of Equation (17) and in the explicit limit evaluated in Section 3.8 rather than in a choice between the two controllers.

### 4.4. Impact of Detailed Non-Ideal Modeling on Efficiency Analysis

The integration of parasitic elements (ESR, DCR) and MOSFET switching parameters ($R_{DS(on)}$, $t_{r}$, $t_{f}$) into the simulation model allowed the principal loss mechanisms to be represented in the efficiency estimate. The steady-state efficiency results, which hovered around 93.6% under full load conditions, are consistent with expected conduction and switching-loss behavior.

### 4.5. Biomimetic Perspective and Contribution

Beyond the numerical trade-offs, the distinguishing feature of the proposed scheme is its explicitly bio-inspired construction. The controller couples two layers of biomimicry that operate at different biological scales: a population-level mechanism, in which swarm intelligence emulates the collective foraging of animal groups to search the parameter space, and an individual-level mechanism, in which the fuzzy reasoner reproduces the linguistic, approximate style of reasoning used by a human expert. Classical tuning approaches—manual, gradient-based, or rule-of-thumb—adjust a controller from the outside; by contrast, the present method uses a biological search principle to reshape the controller’s internal decision boundaries. Figure 6 illustrates the effect of this search for both controller types, converting a sluggish, overdamped response into an agile one; for the FLC this is achieved by the swarm adapting the controller’s perceptual thresholds, in the same spirit that adaptation reshapes an organism’s stimulus–response map. The ablation of Section 3.6 isolates the benefit of this mechanism: with the same optimizer, the same budget and the same number of free parameters, adapting the internal decision boundaries attains a 12.3% lower ITAE cost and a roughly 40% shorter large-signal settling time than rescaling the controller’s inputs, at the cost of a larger peak excursion. In biomimetic terms, adapting the perceptual thresholds is more effective than adjusting sensory gain, which is the specific sense in which the biological analogy carries engineering content here. Positioned this way, the study is a concrete transfer of biological collective and cognitive principles to a power-electronics regulation problem, which is the central concern of biomimetic engineering [44]. It should be emphasized, however, that all results reported here are obtained from a detailed but simulation-only model; the present work is not hardware-validated, and experimental confirmation of the bio-inspired controllers is retained as future work.

### 4.6. Limitations of the Present Study

Several limitations delimit the scope of the conclusions that can be drawn, and are stated explicitly here so that the results are not over-interpreted.

Simulation-only evidence: All results originate from a MATLAB Simulink R2024b model. Although the model incorporates datasheet-derived device parameters, lumped parasitic resistances and switching-loss terms, no hardware measurements were performed. The efficiency figures in Table 6, Table 7 and Table 8 should therefore be read as model outputs under the stated loss assumptions rather than as validated converter efficiencies, and the settling and overshoot figures as model-predicted rather than measured quantities.
Unconstrained inductor current: No inductor-current limit, cycle-by-cycle peak-current protection or synchronous-rectifier reverse-current blocking was represented in the model, and no current term appears in the objective of Equation (8). Both controllers were therefore optimized and evaluated in an unconstrained current regime, and the transient excursions quantified in Section 4.3—up to 62.42 A forward and −23.31 A reverse for the PSO-FLC and 57.94 A and −19.65 A for the PSO-PI, against an 8.21 A nominal full-load design peak—exceed the saturation rating of the selected inductor by a wide margin. This is a major limitation on the transferability of the results to hardware. It applies to both controllers to a comparable degree and is therefore a property of the objective rather than of either parameterization, but it means that the transient comparison of Section 3.5 characterizes the unconstrained response of the two tuned control laws and must not be read as attainable hardware performance. A hardware realization would require a cycle-by-cycle current limit near the inductor rating together with reverse-current blocking and—because such a limiter would act during precisely the transients that the time-weighted criterion rewards—re-optimization under the composite objective of Equation (17). Section 3.8 takes the first of these two steps: with a 10 A cycle-by-cycle limit and reverse-current blocking added to the model, the already-tuned controllers remain stable, the excursions are confined to the band from −0.20 A to 10.21 A and the relative ordering is preserved. That evaluation does not substitute for the second step. Re-optimizing the two controllers under an objective that prices the current violation may place them at different points of the speed–stress trade-off than the unconstrained search did, and establishing where they land remains future work.
Reporting of the stochastic search: Ten independent PSO runs were executed for each controller under a fixed seed sequence (seeds 101–110). The per-run parameter sets, the attained ITAE values and their mean, median and sample standard deviation are reported in Table 3. The dispersion of the attained cost is small for both problems, amounting to approximately 1.1% of the mean for the PI search and 1.4% for the FLC search, which is consistent with the low dimensionality of the two bounded search spaces and indicates that the best-run parameter sets used in Section 3 are representative rather than exceptional. Reporting multi-run distribution metrics in this way follows established practice for stochastic swarm optimizers [45,46]. The per-iteration best-so-far histories of the individual runs are reported in Figure 3, so the characterization covers the convergence behavior of the search as well as the attained final cost. The runs share a single fixed seed sequence and a single disturbance profile, so the dispersion quantifies the sensitivity of the search to its random initialization under that profile and not the sensitivity of the tuned controllers to a change of operating scenario.
Scope of the ablation: The ablation of Section 3.6 separates membership-boundary tuning from scaling-gain tuning under a common budget, but it does not exhaust the design space. The scaling-gain variant was constructed by fixing the membership geometry at the manually tuned baseline of Section 3.1; a different fixed geometry would shift the FLC-B results, so the comparison establishes that boundary tuning is more effective than gain tuning for a conventional starting partition rather than for every possible one. The rule base, the change-of-error partition and the output partition were held fixed in all variants and were not themselves optimized, and the ablation was carried out under the single unweighted ITAE criterion, so it identifies the more effective parameterization for that objective and not necessarily for an overshoot-constrained one.
Single optimizer: Only particle swarm optimization was applied. No alternative metaheuristic was implemented on the same plant, disturbance profile and budget, so the results establish which parameterization of the fuzzy controller the swarm exploits more effectively and must not be read as a statement about the relative merit of PSO among optimization strategies. A benchmark of that kind would require re-implementing the competing optimizers under an equivalent budget, as noted in Section 4.1.
Idealized controller timing: Both controllers were tuned with the control law evaluated at the solver step ($T_{c}=88.9$ ns, fifty samples per switching period), so the optimization itself was carried out against an effectively continuous-time controller. The sensitivity analysis of Section 3.7 shows that re-evaluating the tuned parameters at a realizable update rate ($T_{c}=T_{sw}=4.44\;\mu$s with a zero-order hold) preserves stability and the relative ranking of the two controllers, but computational delay, quantization and sample-and-hold effects were not represented during tuning, and the absolute settling times should be read as optimistic with respect to a digital realization.

## 5. Conclusions

This paper compared a PSO-tuned PI controller with a PSO-optimized fuzzy logic controller for a 153 W, 225 kHz synchronous boost converter regulating a 48 V bus over a 22–30 V input range. The scheme is framed as a two-layer bio-inspired strategy: a swarm-intelligence metaheuristic that mimics collective biological foraging tunes a fuzzy reasoner built on cognition-inspired linguistic reasoning. Its defining feature is that the membership-function limits themselves—the controller’s internal decision boundaries—are placed inside the search space, rather than restricting the metaheuristic to the external scaling gains as is common in the literature [31,32]. Both controllers were tuned with the same ITAE objective, the same swarm size and iteration limit, the same search-space dimension and the same disturbance profile, and were evaluated on a switch-level model incorporating ESR, DCR, $R_{DS(on)}$ and switching-loss terms.

The simulation results support a single, application-dependent conclusion: neither controller dominates, and the two differ in which quantity they protect. The PSO-FLC settles faster in every large-signal input-voltage and reference transition—85 ms against 240 ms for the 24→28 V step, 118 ms against 400 ms for 28→22 V and 174 ms against 380 ms for 22→30 V (Table 6), and 128 ms against 240 ms and 90 ms against 305 ms for the two reference steps (Table 8)—corresponding to reductions of up to 70.5%. The PSO-PI produces the smaller peak deviation in every one of those input-voltage transitions (12.6% against 17.0%, −13.0% against −20.0%, and 26.2% against 40.0%, Table 6) and recovers faster from load steps at the nominal input, 4.0 ms and 2.0 ms against 10.0 ms and 4.5 ms (Table 7). Steady-state behavior is essentially indistinguishable: the full-load inductor-current ripple differs by about 2% (2.047 A against 2.091 A) and the simulated efficiency by no more than 0.07 percentage points (Table 9).

Two findings qualify the faster settling of the PSO-FLC and are, from a design standpoint, the most consequential results of the study. First, both tuned control laws leave the steady-state current envelope by a wide margin and do so to a comparable degree: on the 22→30 V step the peak inductor current reaches 62.42 A for the PSO-FLC and 57.94 A for the PSO-PI, and the 28→22 V step drives both into reverse conduction, to −23.31 A and −19.65 A respectively. Every one of these values exceeds both the 8.21 A nominal full-load design peak of the inductor current and the saturation rating of the selected inductor, and the 7.7% separation between the two peaks is small beside that common margin, so the excursions follow from optimizing an objective without a current term rather than from either controller structure. Adding an explicit 10 A cycle-by-cycle limit confines both controllers to the band from −0.20 A to 10.21 A while preserving their relative ordering Section 3.8 and Section 4.3.

Second, on that same step neither controller as tuned here keeps the output within a standard 60 V/63 V device rating—60.7 V for the PSO-PI against 67.2 V for the PSO-FLC—because the single unweighted ITAE criterion rewards error accumulation and places no penalty on peak excursion (Section 4.2). Deploying either controller over this input range would require higher-rated devices, an explicit derating margin, or a composite objective containing an overshoot term. Re-evaluating the tuned parameters at a realizable control update rate ($T_{c}=1/f_{sw}=4.44\;\mu$s with a zero-order hold) increased the peak overshoot by 3.0–5.3% and the settling time by 4.5–8.0% for both controllers without altering their relative ranking, so the comparison itself survives the reduction of the control update rate to $T_{sw}$. Computational delay and finite arithmetic resolution were not represented, so the absolute settling times reported here remain optimistic with respect to a digital realization (Section 3.7).

One claim is deliberately not made: the study does not report validated converter performance, because no hardware measurements were taken and all figures are model outputs under the stated loss assumptions. Two questions that the initial comparison left open are answered here. The ablation of Section 3.6 shows that, under an identical optimization budget and with the same number of free parameters, tuning the membership boundaries attains a 12.3% lower ITAE cost and roughly 40% shorter large-signal settling times than tuning the external scaling gains, while conceding a larger peak deviation; extending the search to include $G_{du}$ lowers the cost by a further 2.1% at the price of a third search dimension. Read as a best-against-best comparison, that three-parameter partial joint variant is the strongest fuzzy configuration found here, lying 25.4% below the PSO-PI benchmark in mean ITAE against 23.8% for the dimension-matched two-parameter variant reported in detail, so the advantage of the fuzzy parameterization widens once the constraint of equal parameter count is released. The ten-run statistics of Table 3 show that the attained cost varies across independent runs by approximately 1.1% of its mean for the PI controller and 1.4% for the FLC, so the reported parameter sets are representative of the search rather than single fortunate runs.

Within those limits the results are offered as comparative design guidance rather than a universal recommendation. The PSO-FLC is the appropriate choice where rapid adaptation across a wide operating range is the binding requirement and adequate transient voltage and current margin exists at system level; the PSO-PI is preferable where transient voltage containment is the binding constraint. Inductor-current stress does not separate the two, and is addressed by protection and by the objective rather than by the choice of controller. Future work follows in order of priority: experimental validation on the hardware prototype from which the model parameters were taken; the composite objective of Equation (17), carrying explicit peak-voltage and peak-current penalties, which the ablation identifies as the appropriate remedy for the transient-excursion limitation rather than a change of parameterization; re-optimization at the target control update rate under a cycle-by-cycle inductor-current limit; and an extension of the ablation to the rule base and to the change-of-error and output partitions, which were held fixed in all variants evaluated here.

## Figures and Tables

**Table 1:** Keyconverter specifications used in simulation.

Parameter	Value
Rated output power $P_{out}$ (full load)	153 W
Input voltage $V_{in}$	22–30 V
Output voltage $V_{out}$	45–55 V
Switching frequency $f_{sw}$	225 kHz
Switching period $T_{sw}$	4.44 μs
Duty range $D_{min}$–$D_{max}$	0.333–0.600
Inductor *L*	26 μH
Input capacitor $C_{in}$	470 μF
Output capacitor $C_{out}$	1 mF (electrolytic) + multilayer ceramic capacitors (MLCCs)
MLCC bank capacitance	10.1 μF (10 μF + 0.1 μF)
Equivalent output-capacitor ESR	15 mΩ
Inductor DCR	12 mΩ
MOSFET $R_{DS(on)}$	5.5 mΩ
MOSFET $t_{r}+t_{f}$	≈65 ns
Gate-drive voltage	+12 V turn-on/−3 V turn-off
Dead time	100 ns
Body-diode model	Intrinsic body diode enabled; reverse-recovery charge not parameterized separately
Simulation control-update interval $T_{c}$	88.9 ns (=solver step)
FLC input gains $G_{e}$/$G_{\Delta e}$	1/225,000 $s^{-1}$
Duty-command saturation	D∈[0,0.9] (integrator output limit)
Fuzzy duty-rate gain $KG_{du}$	2 $s^{-1}$ (K=1, $G_{du}=2$ $s^{-1}$)
Load model	Constant resistance: 30 Ω for the line and reference tests; 30 Ω↔ 15.05 Ω for the load-step test

**Table 2:** PSO parameters used for controller optimization.

Parameter	Value
Swarm size	20
Max. iterations	100
Cognitive coefficient $c_{1}$	1.5
Social coefficient $c_{2}$	1.5
Inertia weight *w*	0.7

**Table 3:** Statistical evaluation of 10 independent PSO optimization runs under fixed seed sequence (101–110) for PSO-PI and PSO-FLC controllers.

Run No.	Seed	PSO-PI $J_{\mathrm{ITAE}}$	$K_{p}$	$K_{i}$	PSO-FLC $J_{\mathrm{ITAE}}$	${\mathrm{ZE}}_{\mathrm{width}}$ (V)	${\mathrm{PB}}_{\mathrm{sat}}$ (V)
1	101	3.8914	0.7648	19.5184	2.9837	7.423	21.148
2	102	3.8816	0.7812	20.1126	2.9415	7.582	20.452
3 (Best PI)	103	3.8421	0.7778	19.7972	2.9648	7.478	20.898
4	104	3.9538	0.7418	18.9482	3.0212	7.212	21.798
5	105	3.8657	0.7846	19.8814	2.9308	7.528	20.682
6 (Best FLC)	106	3.8988	0.7702	19.6486	2.9154	7.514	20.756
7	107	3.9212	0.7583	19.4112	2.9923	7.348	21.302
8	108	3.8513	0.7787	19.8218	2.9278	7.522	20.712
9	109	3.9818	0.7349	18.7186	3.0447	7.148	22.102
10	110	3.8735	0.7825	19.9098	2.9518	7.485	20.850
Mean	–	3.8961	0.7675	19.5768	2.9674	7.424	21.070
Std Dev *	–	0.0446	0.0175	0.4431	0.0425	0.144	0.526
Median	–	3.8865	0.7740	19.7229	2.9583	7.482	20.874

* Std Dev denotes the sample standard deviation (n−1) over the ten runs listed above.

**Table 4:** Output membership functions of the fuzzy logic controller. The output is a normalized duty-cycle rate command, Δu∈[−1,1], which is scaled by $G_{du}=2$ $s^{-1}$ and integrated to form the duty cycle as given in Equation (13).

Linguistic Output	Type	Parameters
NB	Left-shoulder triangular	[−1,−1,0]
ZE	Triangular	[−0.5,0,0.5]
PB	Right-shoulder triangular	[0,1,1]

**Table 5:** 3×3 Fuzzy rule base (output linguistic value).

e[k]	Δe[k]		
	NB	ZE	PB
NB	NB	NB	ZE
ZE	NB	ZE	PB
PB	ZE	PB	PB

**Table 6:** Simulation performance under input-voltage variation ($V_{ref}=48$ V) with a constant 30 Ω resistive load, corresponding to approximately 1.6 A at the nominal output voltage.

Condition	PSO-PI							PSO-FLC						
	$V_{\mathrm{out}}$	$V_{\mathrm{os}}$	$V_{\mathrm{os}}$	$\Delta V_{\mathrm{out}}$	$\Delta i_{L}$	$T_{\mathrm{st}}$	η	$V_{\mathrm{out}}$	$V_{\mathrm{os}}$	$V_{\mathrm{os}}$	$\Delta V_{\mathrm{out}}$	$\Delta i_{L}$	$T_{\mathrm{st}}$	η
	(V)	(V)	(%)	(Vpp)	(A)	(ms)	(%)	(V)	(V)	(%)	(Vpp)	(A)	(ms)	(%)
24 → 28 V	47.98	6.030	12.56	0.001	1.990	240.0	93.73	47.96	8.141	16.96	0.020	2.035	85.0	93.74
28 → 22 V	47.87	−6.220	−12.96	0.004	1.990	400.0	93.42	47.90	−9.613	−20.03	0.015	2.080	118.0	93.49
22 → 30 V	48.12	12.567	26.18	0.002	1.955	380.0	93.80	48.05	19.181	39.96	0.018	2.067	174.0	93.80

**Table 7:** Simulation performance under load variation, with the load resistance stepped from 30 Ω to 15.05 Ω and back ($V_{in}=24$ V, $V_{ref}=48$ V).

Condition	PSO-PI							PSO-FLC						
	$V_{\mathrm{out}}$	$V_{\mathrm{os}}$	$V_{\mathrm{os}}$	$\Delta V_{\mathrm{out}}$	$\Delta i_{L}$	$T_{\mathrm{st}}$	η	$V_{\mathrm{out}}$	$V_{\mathrm{os}}$	$V_{\mathrm{os}}$	$\Delta V_{\mathrm{out}}$	$\Delta i_{L}$	$T_{\mathrm{st}}$	η
	(V)	(V)	(%)	(Vpp)	(A)	(ms)	(%)	(V)	(V)	(%)	(Vpp)	(A)	(ms)	(%)
30 → 15.05 Ω	47.90	0.100	0.21	0.020	2.047	4.0	93.61	47.90	0.230	0.48	0.030	2.091	10.0	93.56
15.05 → 30 Ω	47.90	0.150	0.31	0.015	2.095	2.0	93.59	47.90	0.100	0.21	0.017	2.125	4.5	93.60

**Table 8:** Simulation performance under reference-voltage variation ($V_{in}=24$ V) with a constant 30 Ω resistive load. In this table $V_{os}$ is expressed as a percentage of the final commanded reference of each step, i.e., $V_{os,\%}=\left(V_{peak}-V_{final}\right)/V_{final}\times 100$, so that the 55 V and 45 V steps are referred to their own targets rather than to the 48 V nominal bus voltage.

Condition	PSO-PI							PSO-FLC						
	$V_{\mathrm{out}}$	$V_{\mathrm{os}}$	$V_{\mathrm{os}}$	$\Delta V_{\mathrm{out}}$	$\Delta i_{L}$	$T_{\mathrm{st}}$	η	$V_{\mathrm{out}}$	$V_{\mathrm{os}}$	$V_{\mathrm{os}}$	$\Delta V_{\mathrm{out}}$	$\Delta i_{L}$	$T_{\mathrm{st}}$	η
	(V)	(V)	(%)	(Vpp)	(A)	(ms)	(%)	(V)	(V)	(%)	(Vpp)	(A)	(ms)	(%)
0 → 48 V (reference start-up)	47.90	0.000	0.00	0.015	2.069	750.0	93.55	47.90	0.590	1.23	0.015	2.122	425.0	93.60
48 → 55 V	54.88	0.000	0.00	0.030	2.346	240.0	95.28	54.87	0.250	0.45	0.030	2.337	128.0	95.29
55 → 45 V	45.10	0.000	0.00	0.015	1.881	305.0	93.60	45.08	−0.100	−0.22	0.020	1.930	90.0	93.64

**Table 9:** Overall dynamic performance and efficiency comparison between PSO-PI and PSO-FLC across all tested operating scenarios.

Performance Metric/Scenario	PSO-PI	PSO-FLC	Dominant Controller/Advantage
Large-Signal Input-Voltage Variations ($V_{ref}=48$ V)			
24 → 28 V Step Settling Time ($T_{st}$)	240.0 ms	85.0 ms	PSO-FLC (64.6% faster recovery)
24 → 28 V Step Peak Overshoot ($V_{os}$)	6.03 V (12.6%)	8.14 V (17.0%)	PSO-PI (Lower voltage stress)
28 → 22 V Step Settling Time ($T_{st}$)	400.0 ms	118.0 ms	PSO-FLC (70.5% faster recovery)
28 → 22 V Step Peak Undershoot ($V_{os}$)	−6.22 V (−13.0%)	−9.61 V (−20.0%)	PSO-PI (Tighter voltage containment)
22 → 30 V Step Settling Time ($T_{st}$)	380.0 ms	174.0 ms	PSO-FLC (54.2% faster recovery)
22 → 30 V Step Peak Overshoot ($V_{os}$)	12.57 V (26.2%)	19.18 V (40.0%)	PSO-PI (Lower peak excursion)
Load Transients ($V_{in}=24$ V, $V_{ref}=48$ V)			
30 → 15.05 Ω Step (Half to Full Load) $T_{st}$	4.0 ms	10.0 ms	PSO-PI (60.0% faster recovery)
15.05 → 30 Ω Step (Full to Half Load) $T_{st}$	2.0 ms	4.5 ms	PSO-PI (55.6% faster recovery)
Load Transient Voltage Deviation ($V_{os}$)	0.10–0.15 V	0.10–0.23 V	Comparable (<0.5% error)
Reference-Voltage Tracking ($V_{in}=24$ V)			
48 → 55 V Step Settling Time ($T_{st}$)	240.0 ms	128.0 ms	PSO-FLC (46.7% faster recovery)
55 → 45 V Step Settling Time ($T_{st}$)	305.0 ms	90.0 ms	PSO-FLC (70.5% faster recovery)
Steady-State Metrics			
Full-Load Inductor Current Ripple ($\Delta i_{L}$)	2.047 A	2.091 A	Comparable (≈2.1% difference)
Conversion Efficiency Range (η)	93.42–95.28%	93.49–95.29%	Equivalent (≤0.07% difference)
Primary Design Suitability	Tighter voltage limits	Fast dynamic adaptation	Application-dependent trade-off

**Table 10:** Ablation study comparing the proposed membership-boundary tuned FLC (FLC-A) against a scaling-gain tuned FLC (FLC-B), a three-parameter partially joint-optimized FLC (FLC-C) and the PSO-PI benchmark. All four variants were optimized under an identical budget: 20 particles, 100 iterations, ten independent runs, and the same ITAE objective, disturbance profile and seed sequence.

Attribute	FLC-A	FLC-B	FLC-C	PSO-PI
	(Proposed)	(Scaling-Gain)	(Partial Joint)	(Benchmark)
Search-space dimension	2D	2D	3D	2D
Tuned parameters	$ZE_{width},PB_{sat}$	$G_{e},G_{\Delta e}$	$ZE_{width},PB_{sat},G_{du}$	$K_{p},K_{i}$
Search bounds	[0,10] V	[0.1,5.0]	[0,10] V	[0,20]
	[0,50] V	$\left[0.1,5\right]\;f_{sw}$	[0,50] V	[0,100]
			[0.5,5.0] $s^{-1}$	
Best parameter vector	7.514 V	1.385	7.620 V	0.7778
	20.756 V	$1.685\times 10^{5}$ $s^{-1}$	19.845 V	19.7972
			2.185 $s^{-1}$	
Attained final cost over ten independent runs				
Mean $J_{ITAE}$	2.9674	3.3850	2.9050	3.8961
$\sigma_{ITAE}$ (n−1)	0.0425	0.0512	0.0415	0.0446
Best $J_{ITAE}$	2.9154	3.3210	2.8410	3.8421
24 → 28 V input-voltage step				
$T_{st}$ (ms)	85.0	142.0	81.0	240.0
$V_{os}$ (V)	8.141	6.850	7.820	6.030
$V_{os}$ (%)	17.0	14.3	16.3	12.6
28 → 22 V input-voltage step				
$T_{st}$ (ms)	118.0	195.0	112.0	400.0
$V_{os}$ (V)	−9.613	−7.920	−9.210	−6.220
$V_{os}$ (%)	−20.0	−16.5	−19.2	−13.0

**Table 11:** Sensitivity of the large-signal dynamic performance to the control update interval $T_{c}$ ($V_{ref}=48$ V, constant 30 Ω resistive load). The solver-step column repeats the values of Table 6; the switching-period column reports the same tuned controllers re-evaluated with a zero-order hold at $T_{c}=T_{sw}=4.44\;\mu$s.

Condition	Controller	$T_{c}=88.9$ ns (Solver Step)			$T_{c}=4.44\;\mu$s (ZOH at $f_{\mathrm{sw}}$)		
		$T_{\mathrm{st}}$	$V_{\mathrm{os}}$	$V_{\mathrm{os}}$	$T_{\mathrm{st}}$	$V_{\mathrm{os}}$	$V_{\mathrm{os}}$
		(ms)	(V)	(%)	(ms)	(V)	(%)
24 → 28 V	PSO-PI	240.0	6.030	12.56	252.0	6.223	12.96
	PSO-FLC	85.0	8.141	16.96	91.0	8.507	17.72
28 → 22 V	PSO-PI	400.0	−6.220	−12.96	418.0	−6.438	−13.41
	PSO-FLC	118.0	−9.613	−20.03	126.0	−10.074	−20.99
22 → 30 V	PSO-PI	380.0	12.567	26.18	398.0	12.944	26.97
	PSO-FLC	174.0	19.181	39.96	188.0	20.198	42.08

**Table 12:** Dynamic performance under the input-voltage profile with an explicit cycle-by-cycle inductor-current limit ($I_{lim}=10$ A) and synchronous-rectifier reverse-current blocking ($V_{ref}=48$ V, constant 30 Ω resistive load). The controllers are those optimized in Section 3; no parameter was re-tuned. The corresponding unconstrained values are given in Table 6 and Section 4.3.

Condition	Controller	$i_{L,\max}$	$i_{L,\min}$	$V_{\mathrm{os}}$	$V_{\mathrm{os}}$	$T_{\mathrm{st}}$
		(A)	(A)	(V)	(%)	(ms)
24 → 28 V	PSO-PI	10.19	0.01	4.94	10.29	355.0
	PSO-FLC	10.19	−0.09	6.78	14.13	120.0
28 → 22 V	PSO-PI	5.13	−0.17	−6.17	−12.85	401.0
	PSO-FLC	10.15	−0.20	−9.56	−19.92	156.0
22 → 30 V	PSO-PI	10.20	1.32	9.62	20.04	328.0
	PSO-FLC	10.21	−0.10	15.28	31.83	220.0

## Data Availability

Data available in a publicly accessible repository. The original data presented in the study are openly available in Figshare at https://doi.org/10.6084/m9.figshare.33158345.

## Abbreviations

The following abbreviations are used in this manuscript:

CCM	Continuous Conduction Mode
DCR	Direct-Current Resistance
ESR	Equivalent Series Resistance
FLC	Fuzzy Logic Controller
GWO	Grey Wolf Optimizer
ITAE	Integral of Time-weighted Absolute Error
MLCC	Multi-Layer Ceramic Capacitor
MOSFET	Metal-Oxide-Semiconductor Field-Effect Transistor
MPPT	Maximum Power Point Tracking
NB, ZE, PB	Negative Big, Zero, Positive Big (fuzzy linguistic levels)
PEM	Proton-Exchange Membrane
PI	Proportional-Integral
PID	Proportional-Integral-Derivative
PSO	Particle Swarm Optimization
PWM	Pulse-Width Modulation
RHPZ	Right-Half-Plane Zero
SEPIC	Single-Ended Primary-Inductor Converter
Vpp	Volts peak-to-peak
